# The circadian clock gene *bmal1* is necessary for co-ordinated circatidal rhythms in the marine isopod *Eurydice pulchra* (Leach)

**DOI:** 10.1371/journal.pgen.1011011

**Published:** 2023-10-19

**Authors:** Zhang Lin, Edward W. Green, Simon G. Webster, Michael H. Hastings, David C. Wilcockson, Charalambos P. Kyriacou

**Affiliations:** 1 Department of Genetics & Genome Biology, University of Leicester, Leicester, United Kingdom; 2 German Cancer Research Center, Heidelberg, Baden-Württemberg, Germany; 3 School of Biological Sciences, Bangor University, Bangor, United Kingdom; 4 MRC Laboratory of Molecular Biology, Cambridge, United Kingdom; 5 Department of Life Sciences, Aberystwyth University, Aberystwyth, United Kingdom; The University of North Carolina at Chapel Hill, UNITED STATES

## Abstract

Circadian clocks in terrestrial animals are encoded by molecular feedback loops involving the negative regulators PERIOD, TIMELESS or CRYPTOCHROME2 and positive transcription factors CLOCK and BMAL1/CYCLE. The molecular basis of circatidal (~12.4 hour) or other lunar-mediated cycles (~15 day, ~29 day), widely expressed in coastal organisms, is unknown. Disrupting circadian clockworks does not appear to affect lunar-based rhythms in several organisms that inhabit the shoreline suggesting a molecular independence of the two cycles. Nevertheless, pharmacological inhibition of casein kinase 1 (CK1) that targets PERIOD stability in mammals and flies, affects both circadian and circatidal phenotypes in *Eurydice pulchra (Ep)*, the speckled sea-louse. Here we show that these drug inhibitors of CK1 also affect the phosphorylation of *Ep*CLK and *Ep*BMAL1 and disrupt *Ep*CLK-BMAL1-mediated transcription in Drosophila S2 cells, revealing a potential link between these two positive circadian regulators and circatidal behaviour. We therefore performed dsRNAi knockdown of *Epbmal1* as well as the major negative regulator in *Eurydice*, *Epcry2* in animals taken from the wild. *Epcry2* and *Epbmal1* knockdown disrupted *Eurydice*’s circadian phenotypes of chromatophore dispersion, *tim* mRNA cycling and the circadian modulation of circatidal swimming, as expected. However, circatidal behaviour was particularly sensitive to *Epbmal1* knockdown with consistent effects on the power, amplitude and rhythmicity of the circatidal swimming cycle. Thus, three *Eurydice* negative circadian regulators, *Ep*CRY2, in addition to *Ep*PER and *Ep*TIM (from a previous study), do not appear to be required for the expression of robust circatidal behaviour, in contrast to the positive regulator *Ep*BMAL1. We suggest a neurogenetic model whereby the positive circadian regulators *Ep*BMAL1-CLK are shared between circadian and circatidal mechanisms in *Eurydice* but circatidal rhythms require a novel, as yet unknown negative regulator.

## Author summary

Molecular feedback loops underlie expression of circadian ~24-hour behavioural and physiological rhythms in terrestrial animals, with PERIOD, TIMELESS and CRYPTOCHROME2 representing the negative, and CLOCK and BMAL1/CYC the positive regulators, with casein kinase 1 (CK1) acting as a modulator. In coastal marine animals, the lunar-mediated ~12.4 hour circatidal rhythm represents the dominant biological cycle but its molecular basis is unknown. The available evidence indicates that circadian and circatidal mechanisms are molecularly independent yet in the speckled-sea louse, *Eurydice pulchra (Ep)*, pharmacological manipulation of CK1 generates correlated changes in both types of cycles, suggesting a shared component. Here, using a Drosophila cell system, we show that CK1 inhibitors alter post-translational modifications of *Ep*CLOCK-BMAL1 leading to a reduction in their transcriptional ability and suggesting that these functional changes in *Ep*CLOCK-BMAL1 may have produced the CK1 inhibitor-mediated circatidal phenotypes. To test this directly we used dsRNAi to knockdown the expression of *Ep*BMAL1 in adult *Eurydice* captured from the wild. We also downregulated the main negative regulator *Ep*CRY2. We observe consistent disruptions to circatidal rhythms in *Ep*BMAl1 but not *Ep*CRY2 knockdowns suggesting that the positive circadian regulator is involved in both circadian and lunar-mediated behaviour.

## Introduction

Circadian clocks are composed of a number of intersected negative feedback loops in which cycling components cycle with ~24 h rhythmicities [1,2]. In higher eukaryotes such as insects and mammals these components are expressed in neurons to mediate circadian behaviour and in peripheral tissues where they control rhythmic tissue and cell-specific functions and metabolism [3,4]. The core negative regulators are PERIOD, TIMELESS or CRYPTOCHROME2, which rhythmically and negatively feedback to suppress the actions of their positive transcription factors, BMAL1 (CYCLE) and CLOCK. There are also a number of kinases and phosphatases that modulate the stability of these regulators, including casein kinase 1 (CK1). *Drosophila melanogaster* has a single gene that encodes or DOUBLETIME (DBT, aka CK1ε), whereas mammals have two circadian-relevant isoforms, CK1ε and CK1δ. Nevertheless, in both flies and mammals CK1 targets the stability of PERIOD proteins [5–7] and determines circadian period length [8–10], thereby highlighting a conserved function.

In contrast to circadian rhythms, the molecular bases of lunar-mediated behavioural and physiological cycles are unknown. Organisms that inhabit the intertidal zone are exposed to the gravitational pull of the moon and the sun on the oceans, so that on most coasts, high/low tide is encountered every 12.4 hours [11]. Animals such as crustaceans are entrained to these environmental cycles but in the absence of tidal stimuli in the laboratory, under constant ‘free-running’ conditions, circatidal (~12.4 h) or circalunidian (~24.8 h) rhythms of behaviour or physiology will persist [12,13]. In addition, semi-circalunar (~15 day) and circalunar (~29 day) rhythms have been observed in a number of intertidal organisms in which life cycle events such as spawning, emergence or reproduction are studied [14,15]. Interactions between the circatidal and circadian clock have also been observed, for example, in determining the period length of marine isopod semi-lunar foraging rhythms [16].

Two main competing hypotheses that attempt to explain how circatidal behaviour could be generated have been presented, including the possibility that there is an independent 12.4 h circatidal oscillator, or that pairs of circalunidian (24.8 h) clocks run in antiphase and evidence has been provided to support both viewpoints, sometime even on the same dataset [17,18]. The problem with many of these studies is that there is no direct evidence at the molecular level to support one over the other competing theories. More recently, knockdown by dsRNAi of both *per* and *Clock* disrupted the circadian modulation of circatidal locomotor activity but not the circatidal period of the mangrove cricket, *Apteronemobius asahinai* [19,20]. A similar result was observed in our previous study of the speckled sea louse, *Eurydice pulchra*, in which knockdown of *Epper* to ~20% of normal levels dramatically disrupted the circadian phenotypes of chromatophore dispersion, *Eptim* mRNA cycling (the only canonical clock component that shows 24 h cycling in *Eurydice*), but left the circatidal locomotor cycle intact [12]. Furthermore, maintaining the sea louse in constant bright light that severely damps the circadian cycles in chromatophore dispersion and *Eptim* mRNA expression had no effect on the period or robustness of circatidal rhythms [12]. Similar conclusions were reached with the circalunar spawning rhythms of the marine polychaete annelid *Platynereis dumerilii* in which pharmacological disruption of circadian clock components with CK1 inhibitors affected circadian molecular and behavioural rhythms but failed to impact the reproductive cycle [15]. Consequently, it would appear that the circadian oscillator as a module does not contribute to core circatidal/circalunar function in at least three higher eukaryotes.

At odds with the conclusion reached above however, and unlike the case with circalunar cycles in *P*. *dumerilii*, treatment of *E*. *pulchra*, with the same CK1 inhibitors generated a dose-dependent lengthening of period for both free-running circadian and circatidal phenotypes [12]. Given that CK1 modulates the stability of PERIOD in flies and mammals [8–10] this result was intriguing because direct dsRNAi knockdown of *Epper* disrupted the circadian but not the circatidal mechanism [12]. We suggested at the time that in *Eurydice*, either CK1 may have several targets [8,21] including an unknown ‘tidal’ protein, or that the inhibitors might be non-specifically disrupting the phosphorylation of an unknown tidally relevant kinase. *D*. *melanogaster* DBT also phosphorylates CLOCK (CLK) [22–24] and CLK stabilises CYCLE (CYC) [25] so a further possibility is that the CK1 inhibitors might have disturbed *Ep*CLK-BMAL1 mediated transcription (BMAL1 is homologous to CYC) and that *Ep*CLK-BMAL1 are required independently for the expression of circadian or circatidal phenotypes.

In this study, we report that these CK1 inhibitors indeed affect *Eurydice Ep*CLK-BMAL1 mediated transcription via the disrupted phosphorylation of both transcription factors, thereby implicating these positive regulators in the CK1-sensitive circatidal mechanism. We therefore directly targeted *Epbmal1* with dsRNAi. In addition, we also knocked down the major *Eurydice* negative regulator *Epcry2* (12). We observe that disruption of the former generates both circadian and circatidal phenotypes whereas *Epcry2* knockdown predominantly affects only the circadian phenotypes. We propose a neurogenetic model that explains how circadian and circatidal phenotypes might be generated.

## Results

### Casein kinase inhibitors inhibit Eurydice CLK-BMAL1 mediated transcription

**Fig 1:**
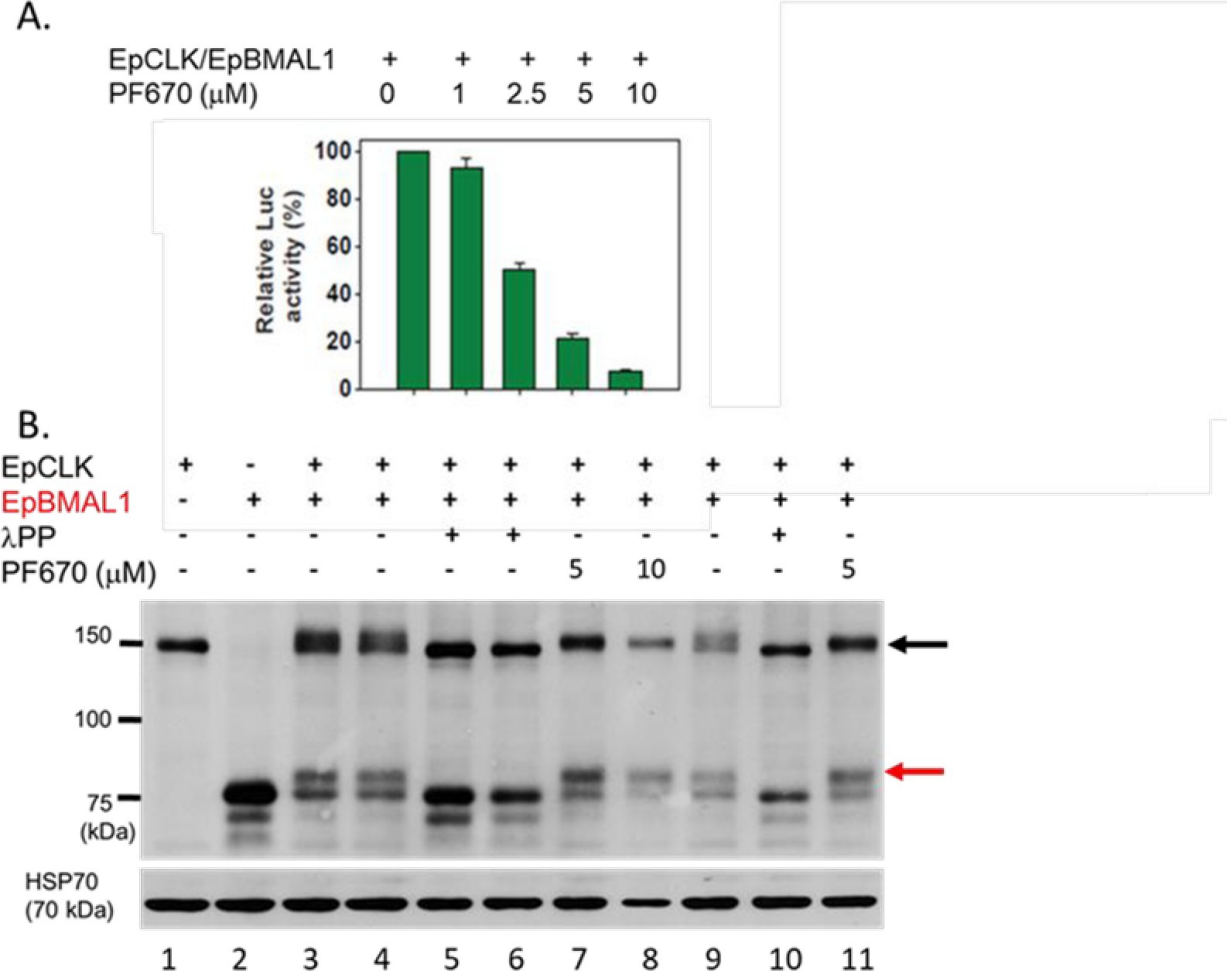
CK1ε/δ inhibitor PF670462 reduces *Ep*CLK/*Ep*BMAL1 E-box mediated transcription by modulating phosphorylation. A. PF670 represses E-box mediated *Ep*CLK-BMAL1 mediated transcription in S2 cells (F_4,10_ = 249.7, p~0, means + sem). B. PF670 alters the phosphorylation profiles of *Ep*CLK (black arrow) and *Ep*BMAL1 (red arrow) in Drosophila S2 cells, λPP lambda protein phosphatase (corresponding Figs for PF480 in S1 Fig).

CLK-BMAL1 heterodimers bind to E-boxes in *per* promoters to transcriptionally activate *per* [26,27] so we utilised the Drosophila S2 cell transcription assay where an E-box containing enhancer is fused to a luciferase reporter [12]. Co-transfection of *EpClk/Epbmal1* gave high levels of luciferase activity [12] (Fig 1A) that were dose-dependently reduced (F_4,10_ = 249.7, p~0) by adding the PF670462 CK1 inhibitor which is more selective for the CK1δ isoform in mammals [21] (Fig 1A). The inhibition of trans-activation occurred in the absence of *Ep*PER (and endogenous *Dm*PER) suggesting that the inhibitors disrupt the phosphorylation of *Ep*CLK-*Ep*BMAL1. S2 cells were transfected singly with either tagged *Epbmal1* or *EpClk*, or co-transfected. Co-transfection revealed a number of additional higher molecular weight isoforms for each corresponding protein in Western blots (Fig 1B). Lambda alkaline phosphatase (λPP) restored each of the bands to their singly transfected original sizes. Administering PF6700462 revealed changes in *Ep*CLK mobility towards the hypophosphorylated isoforms (Fig 1B). Within each lane of the gel, the two *Ep*BMAL1 bands revealed an increase in the relative intensity of the higher MW isoform (Fig 1B red arrow) compared to the lower. This ranged from 47, 47 and 40% without the drug (lanes 3,4 and 9, red arrow, Fig 1B) to 59 and 56% when 5 μM of PF670 were added and 59% with 10μM (lanes 7, 11 and 8 respectively).

We observed an almost identical dose-dependent reduction in transcriptional response in S2 cells with the CK1 inhibitor PF4800567 (F_4,10_ = 169.7 p~0), which is more selective for CK1ε in mammals [21] (S1A Fig). In the corresponding western blot we obtained the same hypophosphorylation of *Ep*CLK at doses of the inhibitor of 5 and 10μM (compare lane 3,4 without inhibitor to lanes 6 and 7). For *Ep*BMAL1 we obtained a similar relative increase in intensity of the higher MW band (61 and 69% with inhibitor compared to 50 and 50% without, lanes 6, 7 compared to 3 and 4 (S1B Fig). Consequently, albeit in a heterologous system, these CK1 inhibitors at different concentrations show consistent effects on the phosphorylation profiles of both *Ep*CLK and *Ep*BMAL1, thereby implicating the positive regulators in the CK1 inhibitor-mediated lengthening of circatidal periods observed previously [12]. We therefore tested for any effects of direct manipulation of *Ep*CLK-*Ep*BMAL1 on circatidal rhythmicity by using gene knockdown.

### Circadian molecular and physiological phenotypes are sensitive to *Epbmal1* and *Epcry2* knockdown

**Fig 2:**
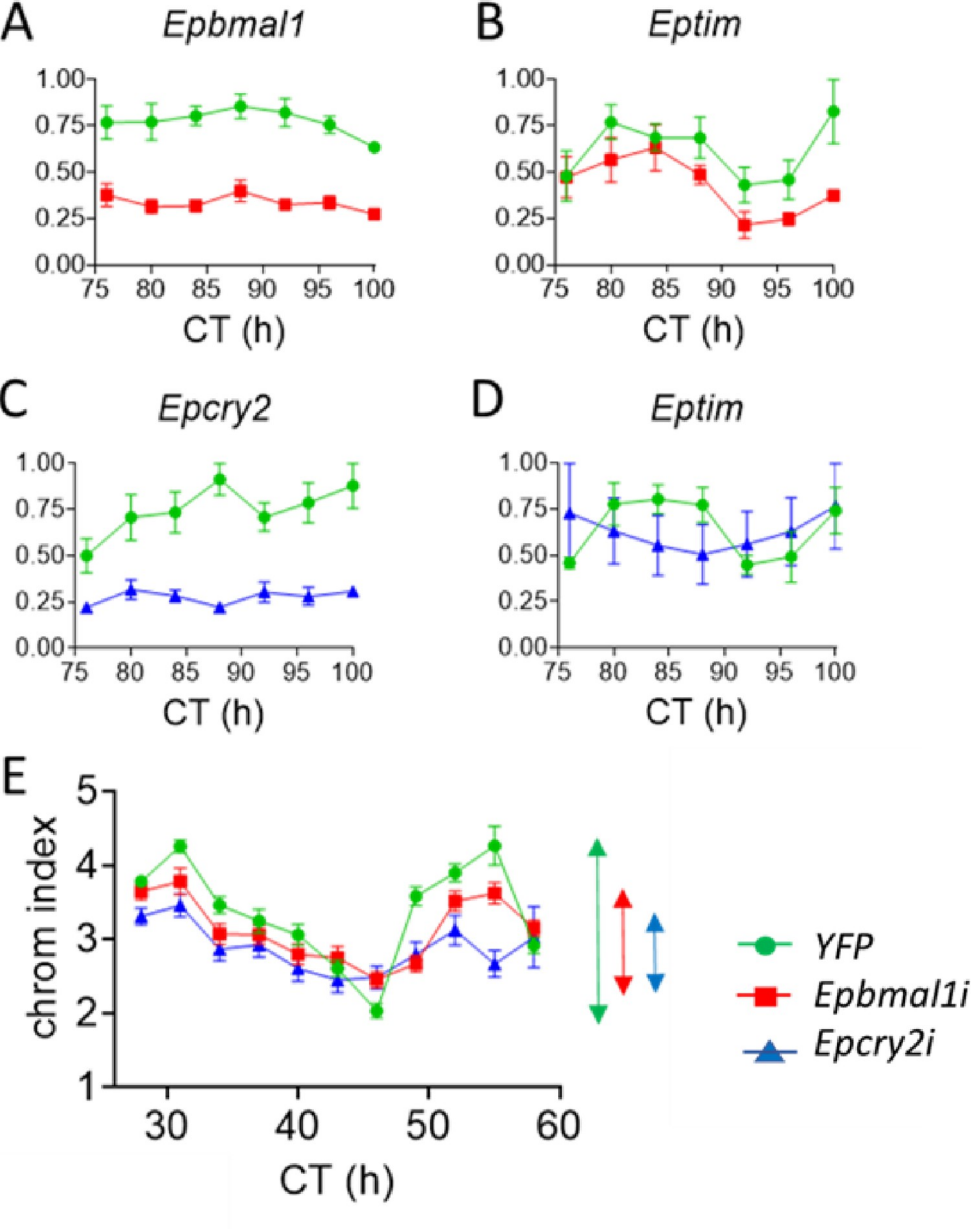
Knockdown of *Epbmal1* disrupts circatidal and circadian phenotypes. A-E. dsRNAi of *Epbmal1* (*Epbmali*, red square) and *Epcry2* (*Epcry2i*, blue triangle) compared to WT^*YFPi*^ control (green circles). A, C. *Epbmal1* and *Epcry2* transcript levels are significantly reduced in *Epbmal1i* (n = 6, F_1,58_ = 154.8), and *Epcry2i* (n = 4, F_1,34_ = 10.38), Knockdown (Knockdown) p = ~0 for both. There are no significant effects of Time. X-axis, circadian time (CT). Y-axis normalised relative abundance. Means +/- sem. B, D. *Eptim* cycles are present in *Epbmal1i* but levels of *Eptim* are significantly reduced. (n = 6, Knockdown F_1,58_ = 11.1 p = 1.5 x 10^−3^, Time F_6,58_ = 4.5 p = 8 x 10^−4^). *Eptim* cycles in *Epcry2i* are altered but there are no significant effects by ANOVA (n = 4). X-axis, CT; Y-axis normalised relative abundance. Means +/- sems. E. Circadian chromatophore cycle. Peak-to-trough amplitudes shown with double-headed arrows on right of panel. ANOVA reveals significant effects for Time (F_10,772_ = 29.7), Knockdown (F_2,772_ = 20.5) and Interaction (F_20, 772_ = 2.82, all p< 4 x 10^−5^). Dunnett’s *post hoc* tests reveals that both *Epbmal1i* (p = 0.0002) and *Epcry2i* (p = 0.00004) are significantly different from *WT*^*YFPi*^ controls. X-axis, CT, Y-axis chromosome dispersion index.

We employed dsRNAi for *in vivo* knockdown of both the positive regulators and, in addition, the potent *Eurydice* negative regulator, *Epcry2* [12]. Exhaustive attempts to reliably reduce *EpClk* levels failed, but a consistent reduction of >50% for both *Epbmal1* and *Epcry2* transcripts was observed in preliminary experiments from the 3/4^th^ day after injection and maintained for several further days compared to controls injected with RNAi to yellow fluorescent protein *(WT*^*YFPi*^*)* (S2A–S2E Figs). *Epcry2* and *Epbmal1* mRNA levels in the control and knockdown animals over the circadian cycle revealed no circatidal or circadian cycling [12] but highly significant reductions to 44% and 43% of control *WT*^*YFPi*^ values were observed for the cognate transcripts respectively (Fig 2A and 2C). In gene-dosage terms, the dsRNAi generates animals that have less than the 50% that would be expected for individuals heterozygous for a wild-type and a null allele for both *Epcry2* and *Epbmal1*. *Eptim* mRNA cycling is observed in *Epbmal1* RNAi *(Epbmal1i)* individuals compared to *WT*^*YFPi*^ during the 4^th^ day of DD but levels of *Eptim* in *Epbmal1i* were, as expected, significantly reduced to 71% of those in *WT*^*YFPi*^ (Fig 2B). The *Eptim* mRNA cycle was dramatically damped in *Epcry2i*, with overall transcript levels at 87% of those in *WT*^*YFPi*^ but there were no significant ANOVA effects due to the small number of replicates in this experiment (Fig 2D).

We observed a clear circadian cycle of chromatophore dispersion in control *WT*^*YFPi*^ animals that was significantly altered in *Epbmal1i and Epcry2i* individuals and reflected in the ANOVA and Dunnett *post hoc* tests with highly significant Time, Knockdown and Interaction factors (Fig 2E). The interaction is generated by the delayed upswing on the second DD cycle of *Epbmal1i* and *Epcry2i* animals which is particularly pronounced in the latter (Fig 2E). The peak-to-trough amplitude index was reduced to ~1–1.25 units for *Epbmal1i* and *Epcry2i* compared to 2.5 for *WT*^*YFPi*^ (Fig 2E). Consequently, dsRNAi of the positive and the negative regulator was effective in altering both the *Eptim* and chromatophore circadian phenotypes.

### Circadian modulation of circatidal behaviour is also sensitive to *Epbmal1* and *Epcry2* knockdown

To analyse the effects of gene knockdown on circatidal swimming, groups of animals were harvested in three main collections on spring tides during full and new moon in the 2016 season. They were injected with dsRNAi constructs after one day and then maintained under constant conditions for 4 days so that their free-running activity recordings were initiated at CT96 (see Methods). The number of animals generating sufficient data for analysis is relatively small for each knockdown within each collection (median = 22).

**Fig 3:**
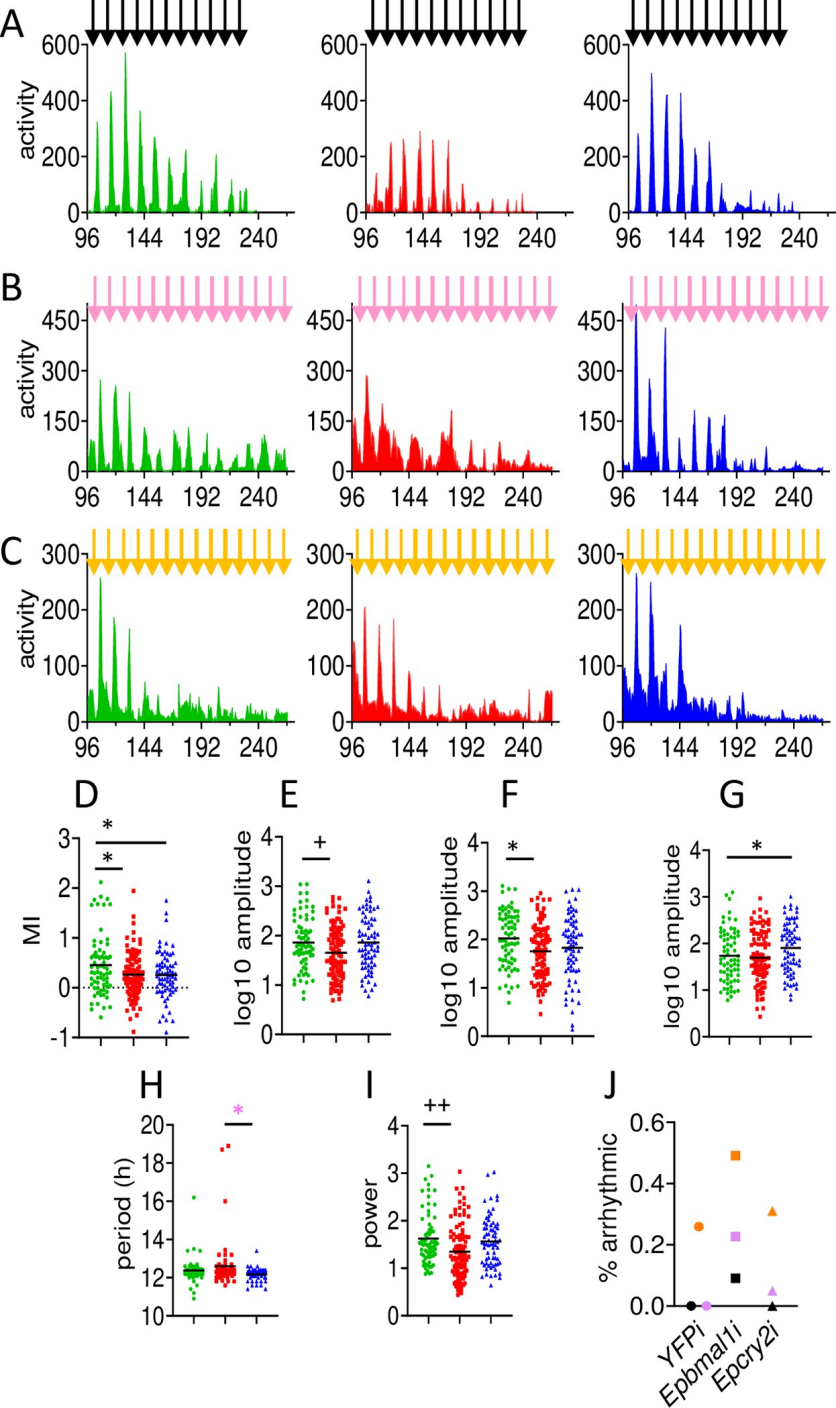
Circatidal rhythms in knockdown animals in season 1 (2016). A-C. Mean activity in 30 min bins of animals collected during spring tides in early summer, new moon (A), midsummer full moon (B) and in late summer/early autumn full/new moons (C). Standard errors of means are omitted for clarity. Panels on left are *WT*^*YFPi*^ (green) middle are *Epbma1i* (red) and right are *Epcry2i* (blue). Vertical arrows represent high tides at Llanddona beach. Animals were collected on the beach, injected with dsRNAi constructs the following day and left for 3 days in DD before being placed in activity monitors to acclimatise for one further day in DD. Activity recording therefore, started at CT96. D-J Statistical analyses of time series in A-C (see S1 Table for all ANOVA results). D. Modulation index E. Overall locomotor amplitude (subjective day plus night locomotor components) in log10 units. F. Night component only G. Day component only H. circatidal period from spectral analysis I. circatidal spectral power J. proportion of arrhythmic animals in each collection for each Knockdown- collection colours correspond to the high tide arrow colours in A-D. * p<0.05, **p<0.01 from two-way ANOVAs and Dunnett’s *post hoc* tests, * from Tukey *post hoc* (see Table 1 and S1 Table). +p<0.05 ++ p<0.01 from one-way pooled seasonal ANOVA. Means shown as horizontal black lines. N for *WT*^*YFPi*^, *Epbmal1i* and *Epcry2i* are in A, 23, 22, 20, B, 20, 22, 20 and C, 27, 57, 29, respectively. Y-axis, locomotor (swimming) events per 30 min time bin. X-axis Circadian Time (CT, h) in constant DD conditions.

Under both laboratory LD cycles and in constant darkness (DD), *Eurydice* show circatidal cycles of swimming in which the night or subjective night component has a higher amplitude than that of the day. This night/day behavioural modulation reflects the expression of the circadian clock and, as expected for arthropods, is absent under constant bright light conditions [12]. It was also absent after *Epper*-targeted RNAi injections in our previous experiments, even in controls, suggesting that the modulation is sensitive to the trauma of this manipulation [12]. It was therefore of interest (and some surprise) that in our current set of injections we observed that the modulation was maintained in most individual animals, and could therefore represent another circadian phenotype by which we can assess the effectiveness of *Epbmal1i* or *Epcry2i* to disrupt the circadian clock. We calculated the modulation index, MI, over the 2016 season from the early, mid and late summer collections from each individual and obtained significant Collection, Knockdown and Interaction terms in two-way ANOVA (see Fig 3 legend and Table 1 for seasonal statistics). Dunnett *post-hoc* tests revealed differences when both *Epbmal1i* (p = 0.037) and *Epcry2i* (p = 0.016) were compared with the *WT*^*YFPi*^ controls (Results of all ANOVAs in S1 Table). This reduction in MI confirms that the circadian component to circatidal behaviour has been disrupted in the two experimental knockdowns. These effects were most pronounced for *Epcry2i* in the early and mid-summer collections, but were not significant in the late summer animals where the MI was severely reduced for the *WT*^*YFPi*^ animals, explaining the significant interaction observed in the ANOVA (S2 Table).

**Table 1:** Circatidal results in two seasons of dsRNAi experiments.

*Knock-down*	*N*	*n*_*arr*_	*% arr*	*Circadian MI*	*Circa-tidal period*, *h*	*Circa-tidal power*	*Circa-tidal amp Day + Night*	*Circa-tidal Night amp*	*Circa-tidal Day amp*	*Seasonal Circatidal Spectral power (Fig 5)*	*Season Autocorrelation (Peak-trough, Fig 5)*			
											*Autocorrelation cycles*			
											*1st*	*2nd*	*3rd*	*4th*
Season 2016														
*WT* ^*YFPi*^	70	7	10.0	0.45 (0.07)	12.38 (0.08)	1.58 (0.07)	1.85 (0.06)	2.01 (0.07)	1.73 (0.07)	3.25	0.91	0.73	0.47	0.39
*Epbmal1i*	101	35	34.6***	0.26* (0.04)	12.59 (0.16)	1.34*++ (0.06)	1.70+ (0.05)	1.73* (0.06)	1.68 (0.06)	1.73	0.40	0.29	0.13	0.16
*Epcry2i*	69	10	14.5	0.26* (0.06)	12.16* (0.04)	1.56 (0.06)	1.90 (0.06)	1.83 (0.08)	1.91* (0.07)	2.40	0.72	0.54	0.36	0.21
**Season 2022**														
*WT* ^*YFPi*^	94	12	12.8	1.03 (0.09)	12.26 (0.05)	1.71 (0.06)	1.62 (0.05)	2.09 (0.04)	1.10 (0.08)	2.04	0.20	0.41	0.16	0.23
*Epbmal1i*	88	26	29.5**	0.76* (0.09)	12.34 (0.05)	1.49* (0.07)	1.56 (0.05)	1.88*++ (0.06)	1.20 (0.07)	1.92	0.24	0.23	0.15	0.15
*Epcry2i*	30	4	13.3	0.6** (0.15)	12.21 (0.08)	1.58 (0.08)	1.71 (0.09)	1.98* (0.08)	1.42 (0.15)	1.96	0.37	0.61	0.22	0.21

The various parameters from the seasonal averages are shown. From two-way ANOVAs *p<0.05, **P<0.01 compared to *WT*^*YFPi*.^ (Dunnett) *p<0.05 *Epcry2i v Epbmal1i* (Tukey). +p<0.05 ++p<0.01 from one way pooled ANOVA–see S2 Table for all F-ratios and *post-hoc* tests. The circatidal spectral peaks and autocorrelation amplitudes (peak R value minus trough) are from Fig 5.

### Circatidal behaviour appears more sensitive to *Epbmal1* than *Epcry2* knockdown

Inspection of Fig 3 reveals that the *Epbmal1i* animals have a generally less coherent and lower amplitude circatidal phenotype than both control *WT*^*YFPi*^ and *Epcry2i*, particularly in the early and mid-summer collections shown in the top two rows of Fig 3. Two-way ANOVA of the overall amplitude (day plus night locomotor component) of the circatidal cycle gave a significant Collection effect (p<0.0001) reflecting a reduction in amplitude in late summer. There was also a marginal Knockdown effect (p = 0.054) whereas seasonal one-way ANOVA pooling the collections for amplitude was significant (p = 0.019) with Dunnett’s *post hoc* comparisons revealing *Epbmal1i* to be significantly different from *YFPi* controls (p = 0.037, S1 Table) reflecting the amplitudes of 1.85–1.90 for *WT*^*YFPi*^ and *Epcry2i* but reduced to 1.70 for *Epbmal1i* (Fig 3E, Table 1). Further analysis of the subjective night locomotor component gave significant Collection (p<0.0001) and Knockdown (p = 0.028) but no Interaction effects, with Dunnett’s generating a significant reduction in *Epbmal1i* amplitude compared to control (p = 0.015 S2 Table, Fig 3F, Table 1). One-way ANOVA confirmed these results (S1 Table). A similar ANOVA for the subjective Day component had significant Collection (p<0.0001) and Knockdown (p = 0.026) but no interaction effects. Dunnett’s revealed an enhancement of Day activity for *Epcry2i* (p = 0.042, Fig 3G, Table 1 and confirmed with one-way ANOVA, S1 Table). Inspection of the mean day and night amplitudes for each collection (S2 Table) shows that the late summer collection gave rather different results from the two earlier ones and included a very high number of arrhythmic animals for all knockdowns, although this was still highest for *Epbmal1i*. In the early and midsummer collections, the main subjective night-time locomotor component was reduced significantly for *Epbmal1i* but not for *Epcry2i* compared to controls, whereas *Epcry2i* but not *Epbmal1i* gave significant elevation of daytime activity. This combination of alterations in locomotor profiles would account for the larger MI reduction for *Epcry2i* (slightly smaller night but larger day component). *Epbmal1i* generates the larger and significant reduction in night amplitude suggesting that this could reflect a direct effect on circatidal amplitude.

We also studied the free-running period in DD and observed a significant Knockdown effect (p = 0.032). Dunnett’s did not reveal any significant differences compared with the *WT*^*YFPi*^ control (means *WT*^*YFPi*^ 12.38 h, *Epbmal1i* 12.62 h and *Epcry2i* 12.17 h) but Tukey generated a significant difference between *Epbmal1i* and *Epcry2i* (p = 0.023), accounting for the ANOVA result (Fig 3H, Table 1, confirmed with one-way ANOVA, S1 Table). We noticed three long period outliers in *Epbmal1i* plus another in *WT*^*YFPi*^ and removing them generated a Knockdown effect that marginally failed to reach significance (p = 0.056, but one way ANOVA, p = 0.038, Fig 3H). We also examined spectral power and observed a significant Collection effect (p<0.0001) but Knockdown failed to reach significance (p = 0.065, yet one-way ANOVA, p = 0.004 with Dunnett’s generating a *YFPi v Epbmal1i* p = 0.005 Fig 3I, Table 1). Finally, we also examined the percentage of arrhythmic individuals within each collection and always observed that *Epbma1i* presented higher proportions of these individuals (Fig 3J, S2 Table). A Fisher exact test on the numbers rhythmic/arrhythmic for the season was highly significant (χ2 = 17.7, df = 2, p = 0.0001, Table 1).

The corresponding spectral and autocorrelation plots for each panel from Fig 3 are illustrated in S3 Fig. While these do not have the resolution of the results based on individuals discussed above, they are nevertheless interesting. In the early and mid-summer collection the spectral analysis reveals a reduced power for *Epbmal1i* compared to *WT*^*YFPi*^ and *Epcry2i*. Similarly, the correlograms show the first four cycles always generating lower peak-to-trough values for *Epbmal1i* compared *WT*^*YFPi*^ and *Epcry2i* (S3A–S3D Fig, S3 Table). In the late summer collection, spectral power is enhanced in *Epcry2i* but the first peak to trough value in the correlogram is highest in *WT*^*YFPi*^ whereas these values in the subsequent peaks are very similar among Knockdowns (S3E–S3F Fig, S3 Table).

To summarise the results for the 2016 season, circadian rhythms of chromatophore dispersion, *Eptim* cycling and modulation of the circatidal cycle are all compromised in *Epbmal1i* and *Epcry2i* animals. Circatidal rhythms are similarly affected in *Epbmal1i* animals with respect to night-time locomotor amplitude, power, proportion of arrhythmic animals and they also show a longer, if rather variable circatidal period whereas *Epcry2i* animals have a slightly shorter period than controls and a higher Daytime locomotor amplitude. Indeed, all the parameters shown in Fig 3 show a significant knockdown effect, although the effect sizes for each were moderate and ranged from 2.6 to 4.4% (S1 Table). These results suggest that *Eurydice* circatidal behaviour is more sensitive to *Epbmal1* than to *Epcry2i* knockdown in spite of the fact that circadian disruption of chromatophore, *Eptim* cycling and the MI index (in two out of three collections, S2 Table) appears more effective in *Epcry2i* knockdowns.

### A second season of injections

**Fig 4:**
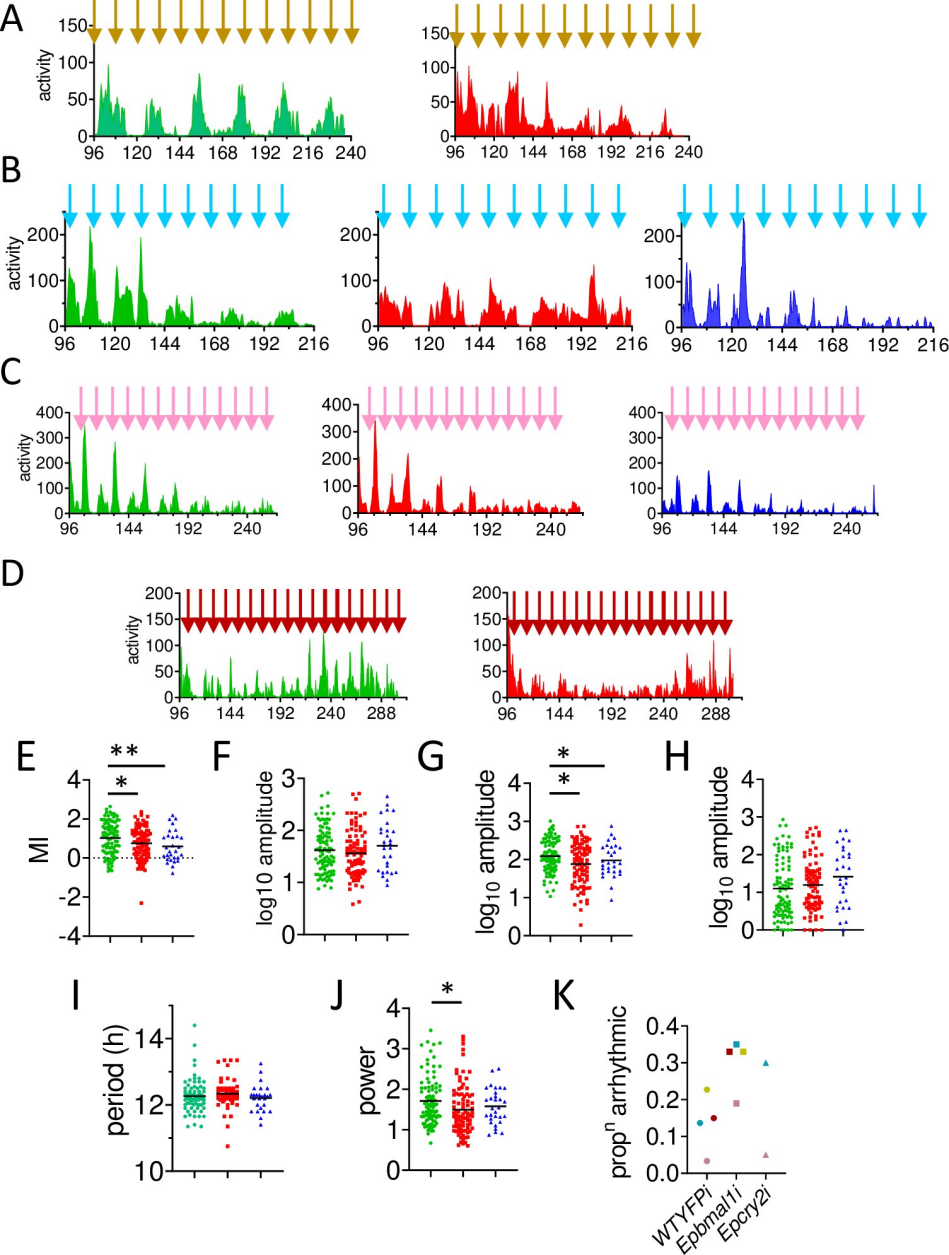
Circatidal locomotor rhythms in knockdown animals in season 2 (2022). As in Fig 3, with locomotor swimming events monitored under constant conditions. Four collections from (A) early summer, new moon (B) mid-summer, full moon(C) late summer, new moon (D) autumn, two collections, full and new moon. In D, animals were maintained in LL after injection. E-K Statistical analyses of time series in A-D (see S1 Table for all ANOVA results). E. Modulation index. F. Amplitude of subjective day and night locomotor components combined (log10 units) G. Night component only H. Day component only I. circatidal period from spectral analysis J. circatidal spectral power K. proportion of arrhythmic animals in each collection for each Knockdown where collection colours correspond to the high tide arrow colours in A-D. * p<0.05, **p<0.01 (from ANOVAs and *post hoc* tests, see text). Means shown as horizontal black lines. Ns for *WT*^*YFPi*^, *Epbmal1i* and *Epcry2i* A; 22 and 18 (no *Epcry2i* animals), B; 22, 17, 10 C; 30, 26, 20 and D; 20, 27 (no *Epcry2i)*, respectively. X-axis, circadian time (CT, h) in constant conditions. Y-axis, locomotor (swimming) events per 30 min time bin.

We repeated our behavioural experiments in the 2022 season using the same dsRNAi constructs. Collections were made in early, mid, late summer with two harvests in the autumn (median number per knockdown per collection = 21). In the early summer, and autumn collections insufficient animals were harvested so we focused on *WT*^*YFPi*^ and *Epbmal1i* injections only (Fig 4A). For the two autumn collections, numbers were also small so they were pooled to provide sufficient data for the same two knockdowns (Fig 4D).

The early summer collection reveals that the *WT*^*YFPi*^ animals are clearly circalunidian, with robust ~25 h cycles of swimming activity with the peak a few hours after the first night-time high tide. In contrast, *Epbmal1i* animals show a mixed profile of circatidal and circalunidian cycles (Fig 4A). In the midsummer collection *WT*^*YFPi*^ animals show discrete ~12 h circatidal cycles which become circalunidian in the latter half of the time series (Fig 4B). The circatidal cycle is also prominent in *Epcry2i* animals whereas the *Epbmal1i* animals generate predominantly lower amplitude circalunidian >24 cycles. The late summer collections all reveal predominantly circatidal cycles which run for 5 days in the *WT*^*YFPi*^ and *Epcry2i* time series whereas this pattern is lost by 3.5 days in *Epbmal1i* (Fig 4C). Finally, the autumn collection which was monitored in constant light (LL) shows discrete circatidal cycles throughout the time series for *WT*^*YFPi*^ animals that is much less apparent in *Epbmal1i* (Fig 4D).

As only two of the four collections included *Epcry2i* animals, two-way ANOVA was initially performed on the *WT*^*YFPi*^ and *Epbmal1i* groups only, with Collection and Knockdown as the main factors. *Epbmal1i* revealed a significant reduction in MI (p = 0.03, Table 1, Fig 4E, S1 and S2 Tables) but no other effects. Overall amplitude (subjective day plus night components) gave a significant Collection (p<0.001), but no Knockdown or interaction effects. As in 2016, there was a significant Knockdown effect on *Epbmal1i* night-time amplitude under both DD and LL that is reduced compared to control *WT*^*YFPi*^ (p = 0.013, S1 and S2 Tables, Table 1, Fig 4G) with no interaction. Daytime amplitude generated a significant Collection (p = 0.022, S1 Table) but no other effects (Table 1, Fig 4H). It would appear that the larger subjective night-time circatidal component is sensitive to *Epbmal1i* knockdown under DD or LL. Analysis of circatidal period revealed that *Epbmal1i* animals have a slight but non-significantly longer period than *WT*^*YFPi*^ (12.32 v 12.25 h) whereas *Epbmal1i* spectral power was significantly reduced compared to *WT*^*YFPi*^ control (p = 0.022, see Table 1, Fig 4I). The latter result is also reflected in the number of rhythmic versus arrhythmic animals (G-test χ2 = 8.98, df = 2, p = 0.01 including *Epcry2i*) with Fig 4K illustrating that in each collection *Epbmal1i* animals generated the highest levels of arrhythmicity.

We extended the two-way ANOVA to the more limited two collections that included *Epcry2i* in which animals were monitored in DD (Table 1). Two-way ANOVA of MI revealed significant Collection (p = 0.002) and Knockdown effects (p = 0.004) in which both *Epbmal1i* and *Epcry2i* generated lower MI values than *WT*^*YFPi*^ with *Epcry2i* showing the largest reduction (Table 1, Fig 4E, S1 and S2 Tables). The night locomotor component was also significant for the Knockdown (p = 0.032) with Dunnett’s revealing a significant difference in *Epcry2i* compared to to *WT*^*YFPi*^ (p = 0.02, S2 Table, Table 1, Fig 4G). Daytime amplitudes showed no significant effects although we note, as in 2016, the *Epcry2i* amplitude was higher than those of the other two groups (Table 1, Fig 4H). There were also no significant differences in circatidal period (S2 Table, Table 1, Fig 4I–4J) but spectral power gave a significant Collection effect (p = 0.003). These two-way ANOVAs on a small subset of the data were buttressed by one-way ANOVAs of all the pooled data for the season. The results are given in S1 and S2 Tables, which confirm knockdown effects on MI, night amplitude and power, with significant decreases for *Epbmal1i* respectively (p = 0.009, p = 0.024).

Spectral plots and autocorrelograms of the overall profiles illustrated in Fig 4 confirm that in the two collections in which *WT*^*YFPi*^ and *Epbmal1i* were compared (early summer and autumn, Fig 4A and 4D), the former shows a very robust behavioural responses as measured by both spectral power and the amplitude of the autocorrelogram cycle (S4A, S4B Fig and S3 Table). In the early summer collection (Figs 4A and S4A, S4B) the spectral and autocorrelation plots reveal that the circalunidian cycle dominates whereas in the autumn (Figs 4D and S4G, S4H) it is the circatidal period that is prominent. The spectral power and autocorrelograms corresponding to the mid-summer collection (Figs 4B, S4C and S4D) confirms the robust circalunidian cycle for *Epbmal1i* whereas in *WT*^*YFPi*^ both circatidal and circalunidian cycles are present and discrete whereas *Epcry2i* shows only a very prominent circatidal cycle. The circatidal cycle also dominates all three Knockdowns in the late summer collection (Figs 4C, S4E, S4F), with *WT*^*YFPi*^ generating the higher values in spectral power and in the autocorrelogram cycle (S3 Table). The autumn LL data shows robust *WT*^*YFPi*^ circatidal spectral plots and autocorrelograms compared to those of *Epbmal1i* (Figs 4D and S4G, S4H and S3 Table).

To summarise the 2022 season results, we observed significant reductions in MI for both *Epbmal1i* and *Epcry2i*, with decreases in circatidal night-time amplitude, rhythmicity and power for *Epbmal1i* animals. Effect sizes were again moderate for the parameters that generated a significant Knockdown and ranged from 2.6 to 8.8% (S1 Table). As in 2016 we also observe small elevations in daytime amplitude for *Epcry2i* in the reduced dataset although this failed to reach significance.

**Fig 5:**
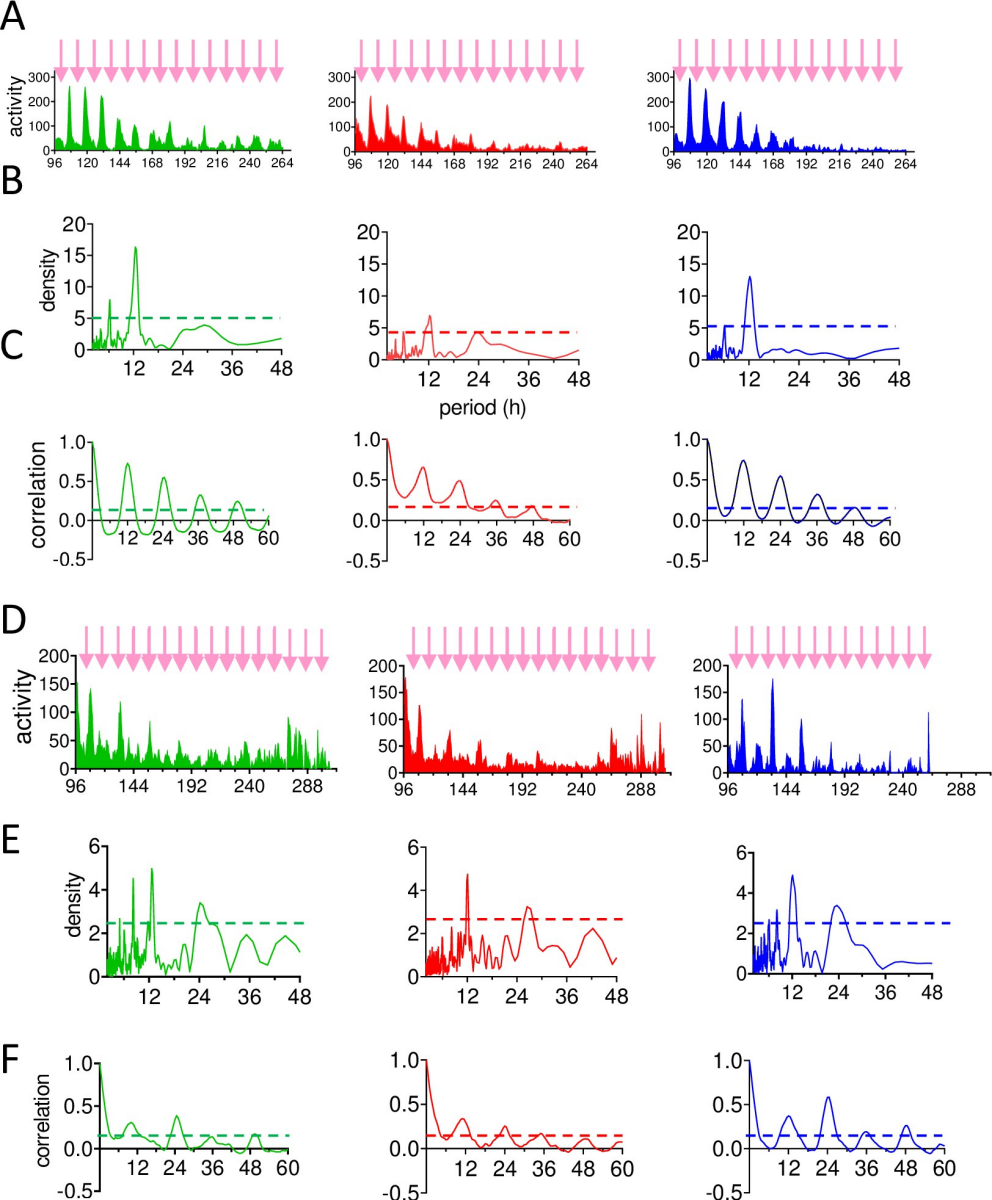
Seasonal circatidal rhythms of dsRNAi knockdowns. A-C season 2016 D-F. season 2022. Collections were pooled for each season and synchronised to the local tidal time. A, D Mean locomotor activity in 30 min bins, arrows denote high tide. X-axis, circadian time (h) in constant conditions. B, E, spectral analysis and C, F autocorrelograms of data in A, D. Dotted lines denote 99% confidence limits. X-axis, period (h).

Fig 5 illustrates the circatidal behaviour resulting from pooling the collections for each season and synchronising them to local tidal time together with corresponding spectral density plots and autocorrelograms. *Epbmal1i* animals show relatively poorly defined circatidal rhythms compared to *WT*^*YFPi*^ and *Epcry2i* in both seasons. The circatidal/circalunidian peak values in the spectral analyses are reported in Table 1 as are the peak-to-trough values from each cycle of the autocorrelograms, which confirm that *Epbmal1i* animals are more sensitive to knockdown than *Epcry2i*.

## Discussion

The link between our previous observation of a dosage-dependent effect on circatidal periods by administering CK1 inhibitors [12] and our approach of knocking down *Ep*BMAL1 and *Ep*CLK, was forged by our results in the S2 cell transcriptional assay. We found that the CK1 inhibitors reduced transcription of an E-box-mediated reporter and that the phosphorylation profiles of *Ep*CLK-*Ep*BMAL1 were altered in the process. We cannot unambiguously state that the inhibitors were directly affecting the phosphorylation of the positive circadian regulators because it is conceivable that the inhibitors may be targeting another kinase non-specifically [21]. Yet irrespective of the kinase identity, changes in the post-translational modifications of *Ep*BMAL1 and *Ep*CLK by the inhibitors indirectly implicated these two positive circadian regulators in the expression of circatidal behaviour.

We therefore employed dsRNAi to knockdown the positive regulator *Epbmal1*, and, as a counterpoint, also the negative regulator *Epcry2* and were successful in reducing the gene dosage of both loci to <50%. In both the *Epbmal1i* and *Epcry2i* animals, gene dosage of the cognate transcripts was reduced to less than that of an animal heterozygous for a wild-type and null mutant allele. For comparison, heterozygous *bmal1/+* mice do not show a significant difference in free-running period nor amplitude compared to wild-type [28,29]. In contrast Drosophila *cyc*^*0*^*/+* heterozygotes show a lengthening of free-running circadian locomotor period of 0.8 h compared to wild-type with no apparent differences in robustness as measured by the proportion of arrhythmic animals [30]. These Drosophila results with *cyc+/cyc*^*0*^ heterozygotes had encouraged us that our >50% knockdown might reveal a circatidal phenotype.

While the knockdowns were not as effective as in our previous study with *Epper*, where we obtained knockdown to ~20% of wild-type levels [12], for both *Epbmal1i* and *Epcry2i*, the knockdowns were sufficient to generate significant changes in circadian phenotypes. From two seasons of collections, in 2016 and 2022, we obtained very similar behavioural results leading us to conclude that the circatidal swimming rhythm of *Eurydice* is more sensitive to reductions in *Epbmal1* dosage than to similar reductions in *Epcry2*. Indeed, the *Epcry2i* knockdown appeared to have a more dramatic effect on both the circadian cycles in chromatophore dispersion and *Eptim* abundance, and generally, a more sustained impact on the circadian modulation of locomotor behaviour as measured by MI in most collections. Yet there was little evidence for any consistent effect on the *Epcry2i* circatidal cycle. While there were marginal changes in circatidal period in both the *Epbmal1i* and *Epcry2i* these were not maintained between seasons (Table 1). The most striking effect was on the robustness of the circatidal rhythm where in both seasons, *Epbmal1* circatidal power and the amplitude of the major night-time locomotor component were significantly reduced, with 29–35% of these animals showing arrhythmicity compared to 10–15% of the *WT*^*YFPi*^ and *Epcry2i* animals (Table 1). These results would suggest a general weakening of circatidal oscillations in *Epbaml1i* animals that is reflected both in the numbers that are arrhythmic and in the major components of the circatidal behavioural cycle.

The results concerning amplitude are somewhat complicated by the circadian modulation of the day/night components. Knockdown of both clock genes reduces the modulation index, MI, for both experimental knockdowns particularly *Epcry2i*. However, in the LL experiment in autumn 2022, the *Eurydice* circadian clock is suppressed [12] so there is little modulation that can be further knocked down and MI values for both *YFPi* and *Epbmal1i* are identical (S2 Table). In both 2016 and 2022 *Epbmal1i* and *Epcry2i* night amplitudes were also consistently reduced compared to *YFPi* implying that the circadian clock normally enhances the night-time locomotor component. However, this suppression is robustly significant for *Epbmal1i* in both seasons and marginally so for *Epcry2i* in 2022 (Figs 3F and 4G, Table 1, S1 and S2 Tables). This additional *Epbmal1i* suppression of night-time amplitude over and above that observed with *Epcry2i* suggests a direct effect of the *Epbmal1i* knockdown on circatidal amplitude rather than an indirect effect of the reduction in circadian modulation.

One curious phenomenon was the switching between circatidal and circalunidian cycles among collections of 2022. In the early and mid-summer collections, the circalunidian cycles were generally more prominent, particularly for *WT*^*YFPi*^ and *Epbmal1i* but not *Epcry2i* (Fig 4A and 4B, S4A–S4C Fig) whereas in the latter two collections of late summer and autumn, the circatidal component dominated (Fig 4C and 4D, S4E to S4H Fig). In early summer the circalunidian component was disrupted in *Epbmal1i* (Fig 4A) whereas in midsummer *Epbmal1i* had prominent circalunidian cycles while *WT*^*YFPi*^ showed particularly discrete circatidal and circalunidian cycles. The major environmental difference between these two collections was that the midsummer one was harvested on a very hot day (15–32°C Night-Day) whereas the early summer harvest was taken under much cooler conditions (9–19°C) although this does help to explain the observation. It simply underscores how little control we have over the environmental conditions underlying the development and behaviour of these animals in the wild.

One caveat to our approach is that unlike gene knockouts, knockdowns do not reduce the targeted gene dosage to zero and so it could be argued that the circadian system in *Eurydice* is simply more sensitive to gene dosage disruptions than the circatidal phenotype. Perhaps then, further reduction in *Epcry2* dosage below 43% might reveal a more striking circatidal phenotype. However, knockdown of *Epper* to ~20% of normal dose that obliterates both the *Eptim* mRNA and the chromatophore circadian cycles, did not alter circatidal behaviour [12]. Nevertheless, it is possible that a complete knockout of *Epcry2*, *Epper* or *Eptim* might generate a circatidal effect. Unfortunately, the long and complex life cycle of *E*. *pulchra* [31] in addition to the difficulty of rearing this species in the laboratory makes a CRISPR/Cas9 gene editing approach impractical. The main advantage to using dsRNAi in the adult is that possible confounding developmental defects of a gene knockout are avoided. For example, in *Drosophila*, *cyc*^*0*^ (and *Clk*^*jrk*^) mutants show reductions in the numbers of pacemaker LNv clock neurons in both adults and larvae as well as abnormal projections from these cells [32,33].

**Fig 6:**
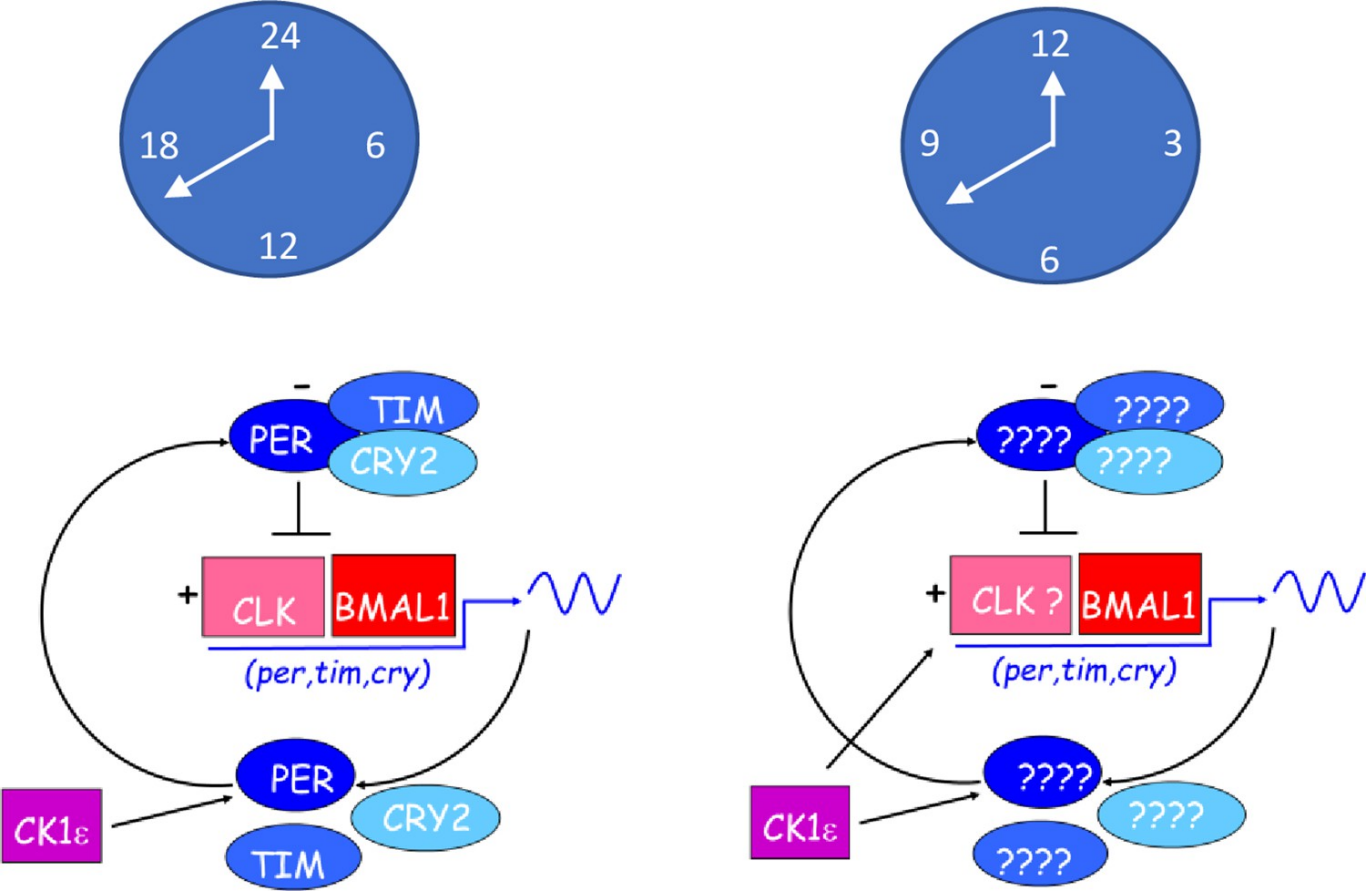
Possible molecular mechanism for circadian and circatidal rhythms in *Eurydice pulchra*. The left-hand panel shows the probable components for the circadian clock as identified in reference [12]. A possible model for the circatidal clock is shown in the righthand panel. BMAL1 (also see ref [36] and CK1ε (and perhaps CLK) are common to both mechanisms but the circatidal negative regulators are unknown at this time.

Our current knockdown results with *Epcry2i* and those of our previous study with *Epper* [12] (that also disrupts the *Eptim* mRNA cycle*)* generate circadian but no consistent circatidal phenotypes, whereas manipulation of *Epbmal1*, affects both types of rhythm. It is therefore tempting to speculate that the negative circadian regulators (*Epper*, *Eptim*, *Epcry2*) may not be involved in the generation of circatidal oscillations, whereas the positive regulator, *Epbmal1* (and possibly *EpClk*) play more fundamental roles. A simple model would have dedicated but separate circadian and circatidal neurons in which all the canonical circadian clock molecules are expressed in the former cells, with cycling *Eptim* expression driving the negative feedback loop (Fig 6). In the latter circatidal cells, the positive regulators EpBMAL1-EpCLK would be present, but not the negative regulators, that would be replaced by a novel circatidal regulator, whose expression would cycle with a ~12 h period and would engage the circatidal negative feedback loop. The two oscillators would interact at the level of output given that *Eurydice* circatidal behaviour show circadian modulation [9]. The model can incorporate a circatidal cycling kinase/phosphatase element driven by the circatidal clock that would feed back onto *Ep*BMAL1-CLK function and support cycling transcription of the novel negative circatidal regulator. This would only occur in circatidal cells, thereby maintaining the separate integrity of the two oscillators.

This model can be tested by extensive investigation of the expression patterns of canonical clock transcripts and proteins both temporally and spatially in the *Eurydice* brain. *Ep*PER appears to be located in a pair of dorsolateral neurons that show circadian cycles of abundance and a further lateral cell that does not [12]. These *Ep*PER neurons are separate from those in *Eurydice* that express PDH [34] which is a marker for brain pacemaker cells in Drosophila [35]. We are currently studying the expression of other clock components with a number of homospecific anti-sera that we have targeted against positive and negative regulators. Our working hypothesis predicts that groups of neurons expressing *Ep*PER, *Ep*CRY2 and *Ep*TIM and *Ep*CLK-EpBMAL1 would represent circadian clock neurons, and neurons expressing the latter positive regulators, but not the negative regulators would define the circatidal neurons.

While we were revising this manuscript we became aware of similar work to ours by Kwiatkowski et al [36]. This study develops the amphipod crustacean *Parhyale hawaiensis* as a circatidal model and uses gene editing to knock out the *Phbmal1* gene, which obliterates circatidal cycles of behaviour in the null mutant. These results are completely consistent with ours. While the *Parhyale* results do not exclude an effect of *Phbaml1i* knockout on the development of circatidal neurons, our work would suggest that the circatidal disruption, even in *Parhyale*, is likely to include a ‘physiological’ effect on the adult. Consequently, the two studies appear to be complementary, and it will be interesting to see in future whether the apparent lack of any robust circatidal effects of *Epper* and *Epcry2* knockdown in *Eurydice*, is matched by corresponding gene knockouts in *Parhyale*. It is notable that the amphipod does not have a *timeless* gene [37], unlike the isopod in which *Eptim* encodes the only robust circadian transcript cycle from the suite of canonical clock genes [12]. Consequently, it is possible that the underlying molecular mechanisms for circadian cycles are different as they are in insects [38], with the implication that the circatidal clockworks may also show some evolutionary flexibility. This view appears to be supported by the observation that knockdown of *Clock* did not impact circatidal cycles in the mangrove cricket [20]. Yet BMAL1 is known to dimerize with other transcription factors apart from CLOCK, so it could be that the circatidal clock uses a different BMAL1-TF combination [39].

In conclusion, it would appear that BMAL1 is a common feature underlying both circadian and circatidal cycles in two crustacean species. Until recently, the literature had reported only the genes that were not required to generate lunar-mediated cycles. From an evolutionary perspective, re-using components for both circatidal and circadian mechanisms would appear to be a pragmatic solution to solving two similar timing problems.

## Materials and methods

### Ethics statement

*Eurydice* are invertebrates so are not subject to licenses for experimentation. Nevertheless, all animals were anaesthetised on ice for injections and similarly anaesthetised before heads were removed for qPCR. Animals were immediately snap frozen for chromatophore analysis.

### Animal collections and behavioral and chromatophore analyses

Animals were netted from Llanddona Beach, Anglesey, North Wales, UK at high water on spring tides (https://www.tidetimes.co.uk/menai-bridge-tide-times-20220620), from June to October and maintained in seawater in LD12:12 at 16°C. In 2016 single collections were made in early and mid-summer, but three collections were required for the late summer group. In 2022, single collections provided sufficient animals for analysis but the autumn collection required two. Swimming activity was recorded in constant darkness (DD) using LAM10 locomotor activity monitor (Trikinetics Waltham MA) at 16°C for 7 days in Sanyo mir254 cooled incubators with white fluorescent light, intensity 485 μW cm^-2^. The late autumn collection of 2022 was maintained in LL but the results were similar to those in DD (see Fig 4D). Animals that showed at least 5 tidal cycles and at least 400 beam interruptions with no more than 5 consecutive half-days (ie subjective night and day) of zero counts over the observation period, were included for further evaluation. For those animals that became inactive towards the end of the experiment and generated a series of ‘0’ counts, the data were trimmed after 12 h of zeros. A few animals showed no activity at the beginning of the experiment but then started moving so these ‘0’ counts at the beginning of the record were also removed. Of the animals injected ~40% did not provide behavioural data for more than 5 circatidal cycles and 400 activity events yet nearly all animals survived to the end of the experiment yet many either did not initiate any locomotor activity or did so before becoming immobile after a few days of sporadic activity. Nevertheless, we were able to produce behavioural data for 65–100 animals per injected Knockdown in each season (except for *Epcry2i* in season 2022, n = 30) that met the selection criteria for time series analyses. Activity events (in 30 min bins) were analysed independently with spectral analysis and autocorrelation [40].

All animals were analysed ‘blind’ to their knockdown ‘genotype’. An animal was considered rhythmic if the two independent time series were significant and generated a consistent period. For the spectral analysis significance meant that the peak in the spectral plot using the CLEAN algorithm was above the 99% confidence limits that were determined using 100 random iterations of the original data. In addition, a significant autocorrelation had also to support the period observed in the spectral plot (statistical protocols described in ref [40]. The power of the rhythm was estimated by taking the peak value of the spectral density plot and dividing by the value of 99% confidence limit. Consequently, a rhythmic animal has a power of >1, but only if confirmed by a significant autocorrelation. Most arrhythmic animals nevertheless showed a peak in the circatidal or circalunidian range that was either not significant by one or other time series analysis, or was non-significant by both. In these cases, the power was obtained by taking the highest value from either the circatidal or circalunidian spectral peak, whichever was the highest, but the period was not used to calculate knockdown values. On rare occasions where there was no obvious peak in the spectral plot, the power at 12.4 h was used. In most animals, the major peak in the spectral plot was in the circatidal range between 12–13 h but the doublet at 24–26 h was also commonly significant. When the power of the doublet was greater than that of the singlet the circatidal period was taken as half of the doublet whereas the power taken was that of the doublet. In the 2016 season collections, 6.8% animals whose activity met the criteria for analysis had doublet>singlet periods whereas in 2022 the doublet>singlets group comprised 32.5%. In one particular collection in early summer 2022 (see Fig 4A), 59% of animals had doublet>singlet. Taking half the doublet as the circatidal period rather than the singlet period has no effect on the overall circatidal period (because the doublet is usually exactly twice the singlet) but it has an effect on the power calculation. In 2022 38%, 31% and 20% of the *WT*^*YFPi*^, *Epbmal1i* and *Epcry2i* animals had doublet>singlet periods, so they are distributed roughly equally among Knockdowns (Fisher exact χ2 = 2.6, df = 2, p = 0.27).

The peak log10 activity value at each subjective night and daytime peak was used as a measure of circatidal amplitude, so each animal generated up to 16 values (representing 8 days of recording) although many animals stopped being active after a few days. The trough value within each successive day or night 12 h segment was nearly always zero so the peak value was taken as the amplitude. For the few animals that did not have a zero in any one or more of their 12 h segments we subtracted the lowest value of activity from the peak for an adjusted amplitude but this had no effect on the results. Adjusted values are also provided in the raw data files. When any 12 h night or day segment contained zero activity a value of 1 was substituted to provide a log10 value of 0. The modulation index, MI was calculated by taking the peak log10 value of activity from each subjective night and subtracting the peak log10 value for each successive subjective day for each animal. To generate the data for Fig 5, the different collections were synchronised to high/low tides.

Two-way ANOVAs were performed on the data for each season with Collections and Knockdowns as the main effects. While there was an *a priori* expectation that the two knockdowns would be different from controls, we nevertheless used the conservative Dunnett *post-hoc* test to compare each experimental group with the *YFPi* controls. In two cases (S1 Table) cases the Knockdown effect was significant in the ANOVA, but the Dunnett test was not. This was because the *Epbmal1i* v *Epcry2i* comparison was contributing to the significant F-ratio (detected with a Tukey test). As sample sizes within each collection was small with a median size of 21–22, we also pooled the data for each genotype for the season and performed one-way ANOVA. All ANOVA results from the behavioural analyses are also presented in S1 Table. Effect sizes were calculated using η^2^. Departures from normality were tested in Prism by default using Anderson-Darling, D’Agostino-Pearson, Shapiro-Wilks and Kolmogorov-Smirnov tests. Nearly all parameters studied passed at least two (and usually all 4) tests. In a few cases where there was significant departure from normality, Kruskal-Wallis ANOVA was used and compared with the corresponding one-way parametric ANOVA. In all such cases both types of ANOVAs generated consistent main effects.

To assess chromatophore rhythms, animals were snap frozen in liquid nitrogen at defined tidal and circadian times, chromatophore patterns imaged by digital camera and scored ‘blind’ using the Hogben and Slome 5-point index [41] which was modified to include 0.5 point scoring intervals [12]. These animals were maintained in LD12:12 for 3 days post dsRNAi injection after which they were placed in DD, and scored every 3 h DD for 30 h during day 4–5 (from the second day of DD). Between 4–6 animals were scored for each time point from each collection. Heads were cropped and snap frozen for later qRT-PCR.

### dsRNAi

Double-stranded RNA (dsRNA) molecules of *EpClk*, *Epbmal1* and *Epcry2* were designed with the E-RNAi web-service [42] and synthesised by using a MEGAscript RNAi kit (Ambion, UK) (S4 Table). For the dsRNAi control, the yellow fluorescent protein (YFP) gene from pEYFP-N1 (Clonetech, UK) was used. 200-250ng of dsRNAs was injected into the hemocoel using air pressure microinjection via glass microcapillary [43]. Gene suppression was assessed by real-time quantitative RT-PCR. *Epbmal1i* animals tolerated the injections rather better than the other two Knockdowns, so numbers surviving to full analysis were greater (see text).

The full length of *EpClk* (NCBI: KC885973), *Epbmal1* (NCBI: KC885968) and *Epcry2* (NCBI: KC885970) subcloned in pAc5.1/V5-hisA vector [12] were used as templates for the target sequences amplification (600bp for *Clk*, 587bp and 570bp for *bmal1*-1 and *bmal1*-2, respectively, 650bp for *cry2*). PCRs were primed using oligonucleotides containing a T7 phage promoter region (S4 Table). Single stranded cRNA in both directions was synthesised and complementary RNA strands were hybridised and purified according to the manufacturer’s instructions. Double stranded products were analysed in agarose gels and concentrated by ethanol precipitation to 3μg/μl in nuclease-free water, aliquoted and kept at -80°C until use. For the dsRNAi control, the yellow fluorescent protein (YFP) encoding gene from pEYFP-N1 (Clonetech, UK) was used to generate a 400bp dsRNA with the same method described as the target sequences. Double-stranded RNA mixed with equal volume of 2x injection buffer (0.2mM sodium phosphate buffer pH 6.8, 10mM KCl) containing filtered food colour was injected into the haemocoel between anterior tergites through a glass microcapillary and using compressed nitrogen/air delivered by a PV830 PicoPump (World Precision Instruments, Inc). Animals were immobilised by leaving on ice and then transferred onto the ice-cold aluminium block using a sieve/mesh for injection under a microscope. About 130-160nl (200-250ng) of dsRNA was injected in each animal. Injected animals were placed on tissue for 2–3 minutes to ensure injected fluids did not leak out of the puncture wound [43]. Gene suppression was assessed by qRT-PCR.

Initial collections for attempting dsRNAi and follow-up qPCR for *EpClk*, *Epcry2*, and *Epbmal1* were made in 2014 and 2015. Once the conditions were optimised, the dsRNAi experiments with *Epbmal1* and *Epcry2*, were performed from collections made from June to September 2016 and repeated from July to October 2022 (in 2020 and 2021 the pandemic had prevented us from travelling to Wales to harvest the animals). Animals collected from the beach were transferred immediately to Leicester where they were injected the following day with dsRNAi constructs (*WT*^*YFPi*^, *Epbmal1i* or *Epcry2i*). The injected animals for locomotor recordings were then placed in DD for 3 further days, then placed in activity monitors for another day in DD to acclimatize, before the Trikinetics monitors were switched on (again in DD). Consequently, the activity recordings begin on the 5^th^ day after injection.

### Real-time quantitative RT-PCR

We examined the expression of *Epbmal1*, *EpClk* and *Epcry2* by qPCR to study the extent of the knockdown as well as to observe the effects on the *Eptim* mRNA circadian cycle [12]. Animals were maintained for different numbers of days in DD for the dsRNAi to take effect and initially mRNA was harvested from 5 animals, 10 h into subjective day (CT10, CT34, CT58 etc) at each day (S2A Fig). The *Eptim* mRNA circadian cycle of expression was quantified by qPCR during the 4^th^ day of DD by taking samples every 4 h.

Total RNA was extracted from pooled heads using Trizol Reagent in conjunction with the PureLink RNA Mini Kit (Invitrogen). DNA contamination was removed by on-column PureLink DNase treatment (Invitrogen). 0.5–1μg total RNA from each sample was used for cDNA synthesis by using the High Capacity cDNA Reverse Transcription Kit (Applied Biosystems) and oligo-dT [12–18] primer (Invitrogen) in a 20μl total reaction volume for 120 minutes at 42°C and the reaction was terminated by heating to 85°C for 5 minutes.

Quantitative PCR was performed on the Roche LightCycler 96 instrument by using GoTaq qPCR Master Mix (Promega) with 1μl cDNA template and 0.5μl of 10mM each primer in a total volume of 25μl reaction. The cycling conditions were as follows: 95°C for 120 seconds, 40 cycles of 95°C for 15 seconds and 60°C for 60 seconds, and then followed by melting curve reaction at 95°C for 10 seconds, 65°C for 60 seconds and 97°C for 1 second. The primer pair for each gene was designed to amplify 100-130bp PCR products (S4 Table). The relative quantification method from Roche LightCycler software was used to calculate gene expression and ratio error. The Standard curves were obtained using decimal dilution series of plasmid DNA. Transcript levels were normalised to the *Eurydice* ribosomal protein L32 gene *(RPL32)* and for *Eptim* mRNA experiments, values were scaled to the timepoint with the highest expression level in *WT*^*YFP*^ controls to allow pooling of biological replicates and statistical analysis by ANOVA. *bmal1*-1 and *bmal1*-2 sequences were equally efficient for knockdown (S2C–S2E Fig) and so *bmal1-1* was used for the injections.

### CK1 inhibition in S2 cells

The *Drosophila* S2 cells (Invitrogen) were maintained in HyClone SFX-insect medium (Thermo Scientific) supplemented with 10% fetal bovine serum (FBS) and penicillin-streptomycin antibiotics at 25°C as described previously [12]. Cells were transfected with expression constructs by using Cellfectin (Invitrogen) according to the manufacturer’s instructions. *EpClk*, *Epbmal1*, were amplified from their corresponding plasmids and sub-cloned into the *Drosophila* S2 cell expression vector pAc5.1/V5-HisA (Invitrogen) as reported previously [12]. Control transfections, including only reporter construct and empty vector (pAc5.1/V5-hisA) established baseline activity. Luciferase activity was measured using the Dual Luciferase Reporter Assay Kit (Promega) and was normalised for transfection efficiency using a *Renilla* expression plasmid. At least three independent transformations were performed for each assay.

CK1ε/δ inhibitor, PF670 or PF480 (Tocris Biosciences) solution was added into S2 cells after 5–6 h transfection to a final concentration as indicated and the drug treated cells were incubated for 48 h at 25°C before harvest for luciferase activity [12] or western analysis. The lambda protein phosphatase treatment is described in the western blot analysis below.

### Western blot

Transfected cells were washed with ice-cold PBS, pelleted at 4°C and lysed in the RIPA buffer (Sigma) along with complete protease inhibitor cocktail (Roche) and PhosSTOP Phosphatase Inhibitor (Roche). For the protein phosphatase treatment, cells were lysed as described above except in the absence of phosphatase Inhibitor and incubated with 400u lambda protein phosphatase (New England Biolabs) at 30°C for 1 h. About 50ug total protein from cell extracts were blotted and hybridised with the primary antibody of Mouse anti-V5 (Invitrogen) for CLK and BMAL1 expression then the secondary antibody of horseradish peroxidase-conjugated either anti-mouse or anti-rabbit IgG antibody (Sigma). Chemiluminescence detection was performed by using ECL Western Blotting detection Reagent (GE Healthcare). HSP70 was used as a general loading control but was not used in the quantification of the BMAL1 hypo/hyperphosphorylated isoforms which were quantified relative to each other using ImageJ software. Three different gels were run for the S2 cell westerns (biological replicates) with multiple lanes carrying both technical but also biological replicates, the latter treated with different drug doses.

## Supporting information

S1 Fig CK1ε/δ inhibitor PF480 reduces EpCLK/BMAL1 E-box mediated transcription by modulating phosphorylation. (PDF)

S2 Fig Preliminary dsRNAi knockdown experiments for *EpClk*, *Epbmal1* and *Epcry2*. (PDF)

S3 Fig Spectral plots and autocorrelograms for knockdowns in 2016 by collection. (PDF)

S4 Fig Pectral plots and autocorrelograms for knockdowns in 2022 by collection. (PDF)

S1 Table Results of behavioural ANOVAs. (PDF)

S2 Table Mean values for circatidal parameters for each collection per season. (PDF)

S3 Table Description of spectral and autocorrelogram plots from S3 and S4 Figs. (PDF)

S4 Table Primers for dsRNAi and qRT-PCR. (PDF)
