# Correction: The circadian clock gene *bmal1* is necessary for co-ordinated circatidal rhythms in the marine isopod *Eurydice pulchra* (Leach)

**DOI:** 10.1371/journal.pgen.1011047

**Published:** 2023-11-15

**Authors:** Lin Zhang, Edward W. Green, Simon G. Webster, Michael H. Hastings, David C. Wilcockson, Charalambos P. Kyriacou

The first author’s name is spelled incorrectly. The correct name is: Lin Zhang. The correct citation is: Zhang L, Green EW, Webster SG, Hastings MH, Wilcockson DC, Kyriacou CP (2023) The circadian clock gene *bmal1* is necessary for co-ordinated circatidal rhythms in the marine isopod *Eurydice pulchra* (Leach). PLoS Genet 19(10): e1011011. https://doi.org/10.1371/journal.pgen.1011011
