# Supplementary material for: The circadian clock gene bmal1 is necessary for co-ordinated circatidal rhythms in the marine isopod Eurydice pulchra (Leach)
Source: PLoS Genet. 2023 Oct 19;19(10):e1011011. doi: 10.1371/journal.pgen.1011011 (PMC10617734; doi:10.1371/journal.pgen.1011011)

S1 Fig. CK1ε/δ inhibitor PF480 reduces *Ep*CLK/BMAL1 E-box mediated transcription by modulating phosphorylation

A. PF480 represses E-box mediated *Ep*CLK-BMAL1 mediated transcription in S2 cells ($F_{4,10}$=169.7 p~0, means + sem).

B. PF480 alters the phosphorylation profiles of *Ep*CLK (black arrow) and *Ep*BMAL1 (red arrow) in Drosophila S2 cells, λPP lambda protein phosphatase. Relative intensities of the two *Ep*BMAL1 isoforms were quantified with Image-J software within each lane, so did not require running a HSP-70 loading control.

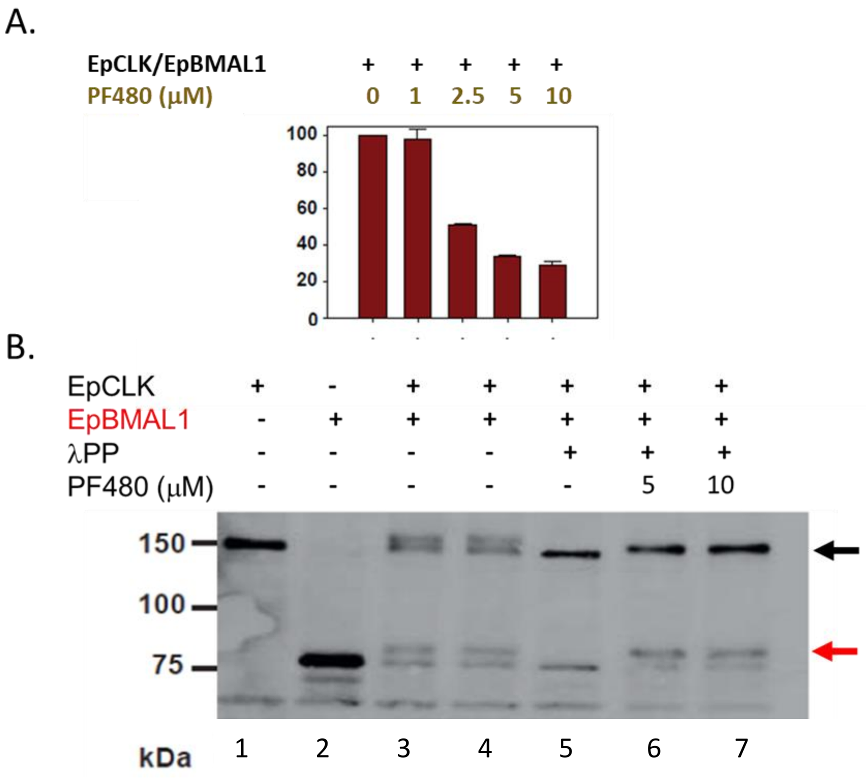

Supplement: S1 Fig — (PDF) [file pgen.1011011.s001.pdf]
