# Supplementary material for: The circadian clock gene bmal1 is necessary for co-ordinated circatidal rhythms in the marine isopod Eurydice pulchra (Leach)
Source: PLoS Genet. 2023 Oct 19;19(10):e1011011. doi: 10.1371/journal.pgen.1011011 (PMC10617734; doi:10.1371/journal.pgen.1011011)

S2 Fig. Preliminary dsRNAi knockdown experiments for *EpClk, Epbmal1* and *Epcry2*

A, B. Two experiments attempting to knockdown *EpClk*. qPCR performed up to 6 days after injection. No consistent long-lasting knockdown was achieved. Means +/- ratio error was calculated using Roche LightCycler software

C, D. Two experiments using two different sequences of *Epbmal1* (F1 and F2) show significant knockdown from days 4-9.

E. Knockdown of *Epbmal1* (F1 sequence) and Ep*cry2* shows significant knockdown at days 3 and 4 after injection (see also Fig 2A-D). Data from combing separate experiments on *Epbmal1F1* and *Epcry2* knockdown by setting YFP values to 100%.

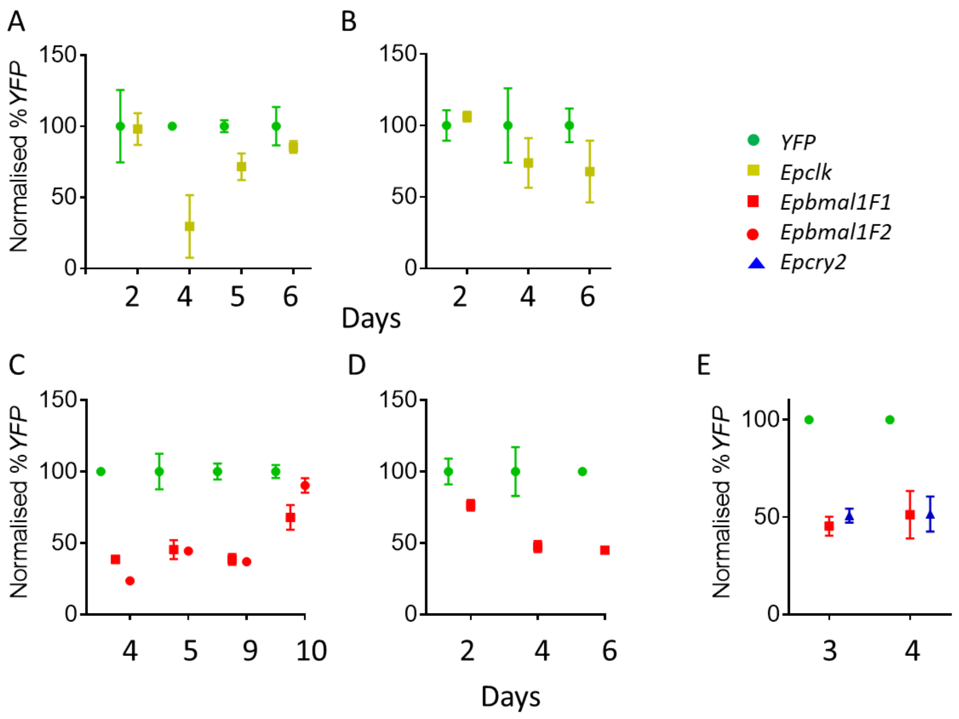

Supplement: S2 Fig — (PDF) [file pgen.1011011.s002.pdf]
