# Supplementary material for: The circadian clock gene bmal1 is necessary for co-ordinated circatidal rhythms in the marine isopod Eurydice pulchra (Leach)
Source: PLoS Genet. 2023 Oct 19;19(10):e1011011. doi: 10.1371/journal.pgen.1011011 (PMC10617734; doi:10.1371/journal.pgen.1011011)
Supplement: S3 Fig — (PDF) [file pgen.1011011.s003.pdf]

S3 Fig. Time-series analyses of *WT*^{YFPi} (green) *Epbmal1i* (red) and *Epcry2i* (blue) knockdowns of 2016 season

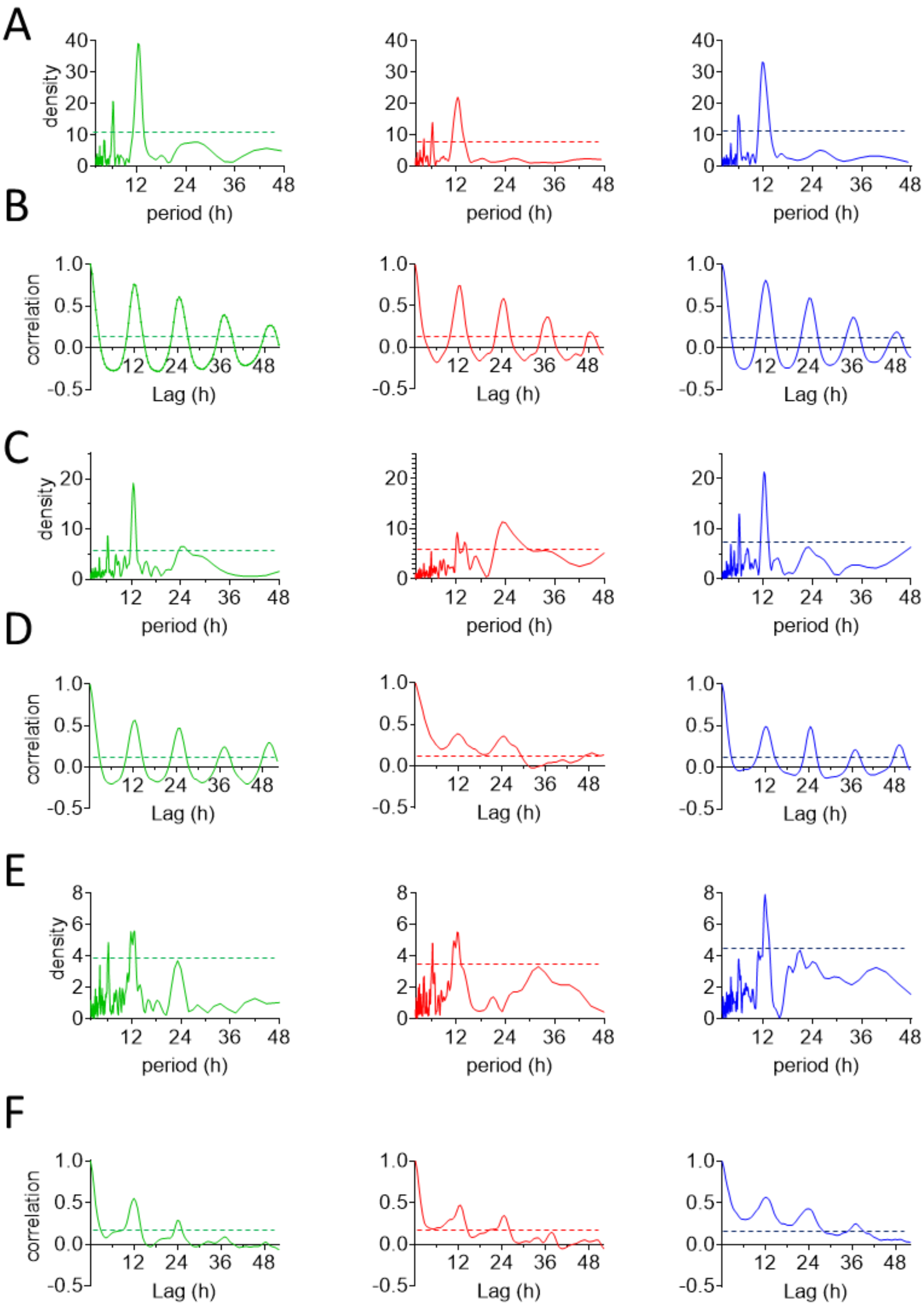

Each panel represents the time series analyses of the locomotor activity profiles from Figs 3A-C (main text). A, B are spectral and autocorrelation analyses of Figs 3A, C D and EF are corresponding analyses of Figs 3B and 3C respectively. The 99% confidence limits for each analysis are shown as dotted lines. The power of the circatidal spectral peak and the amplitude of the autocorrelogram at each cycle are shown in S2 Table.
