# Supplementary material for: The circadian clock gene bmal1 is necessary for co-ordinated circatidal rhythms in the marine isopod Eurydice pulchra (Leach)
Source: PLoS Genet. 2023 Oct 19;19(10):e1011011. doi: 10.1371/journal.pgen.1011011 (PMC10617734; doi:10.1371/journal.pgen.1011011)
Supplement: S4 Fig — (PDF) [file pgen.1011011.s004.pdf]

S4 Fig. Time-series analyses of *WT*^{YFPi} (green) *Epbmal1i* (red) and *Epcry2i* (blue) knockdowns of 2022 season.

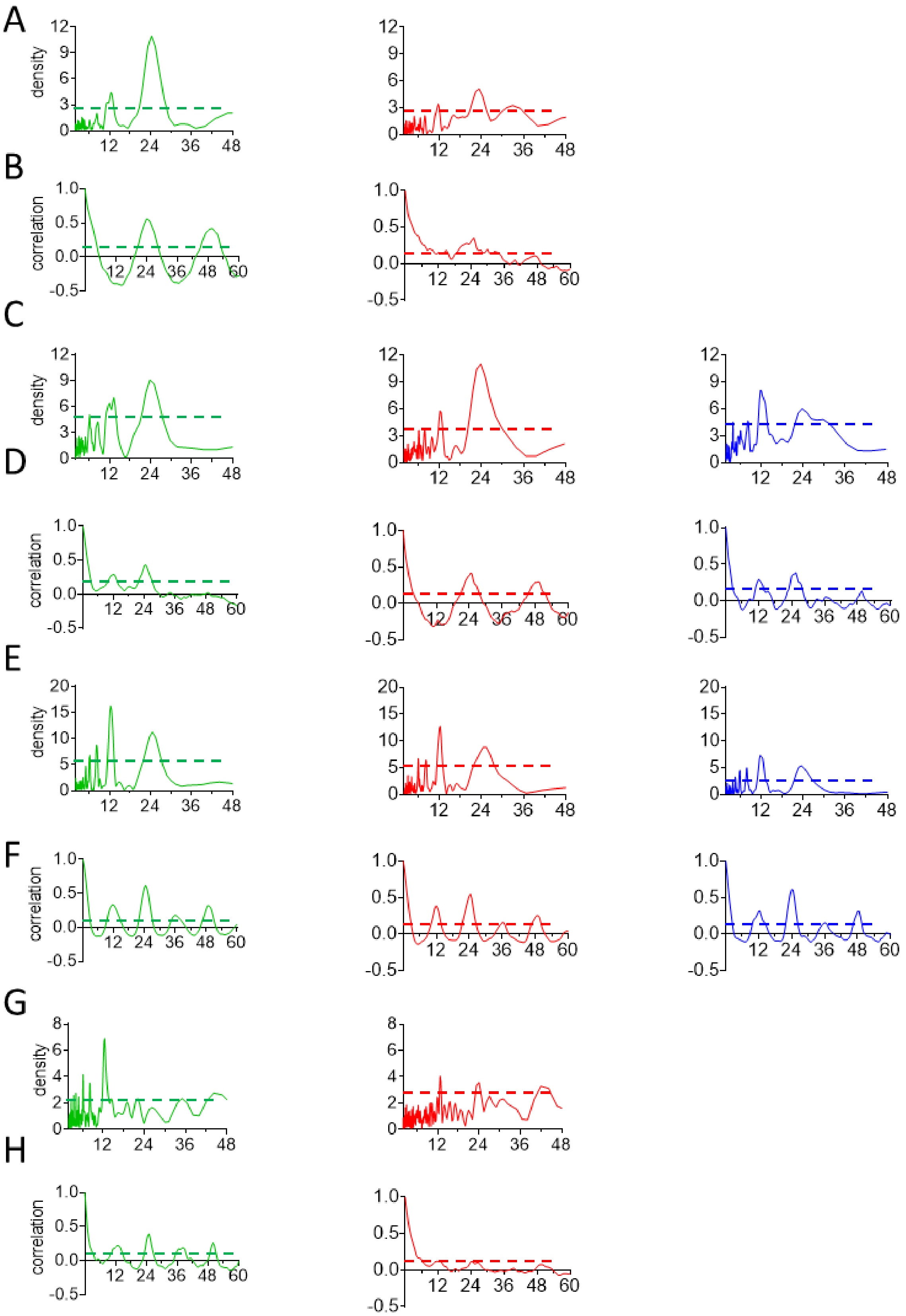

Panels represent the time series analyses of the locomotor activity profiles from Figs 4A-D (main text). A, B are spectral and autocorrelation analyses of Fig 4A. C,D, E,F and G,H are corresponding analyses of Figs 4B, 4C and 4D respectively. The 99% confidence limits for each analysis are shown as dotted lines. The power of the circatidal spectral peak and the amplitude of the autocorrelogram at each cycle are shown in S2 Table.
