# Supplementary material for: The circadian clock gene bmal1 is necessary for co-ordinated circatidal rhythms in the marine isopod Eurydice pulchra (Leach)
Source: PLoS Genet. 2023 Oct 19;19(10):e1011011. doi: 10.1371/journal.pgen.1011011 (PMC10617734; doi:10.1371/journal.pgen.1011011)
Supplement: S1 Table — (PDF) [file pgen.1011011.s005.pdf]

2016	Two-way ANOVA			*post hoc, p (Dunnett)*		*One-way ANOVA*	*post-hoc (Dunnett)*	
	Knockdown, F (p, $\eta 2$)	Collection F (p, $\eta 2$)	Interaction F (p, $\eta 2$)	*Epbmal1i*	*Epcry2i*	Knockdown (p)	*Epbmal1i*	*Epcry2i*
MI	$F_{2,231}$ =4.18 (0.017, *0.034*)	$F_{2,231}$=6.19 (0.002, *0.051*)	$F_{4,231}$=3.26 (0.017, *0.053*)	0.037	0.016	$F_{2,237}$=3.54 (0.031)	0.029	0.051
Amplitude Day + Night	$F_{2,231}$ =2.95 (0.054, *0.025*)	$F_{2,231}$=27.1 (<0.0001, *0.19*)	$F_{4,231}$=0.87 ns	0.14	0.85	$F_{2,237}$=4.05 (0.019)	0.037	0.992
Amplitude Night	$F_{2,231}$ =3.63 (0.028, *0.031*)	$F_{2,231}$=32.1 (<0.0001, *0.217*)	$F_{4,231}$= 1.44 ns	0.015	0.182	$F_{2,237}$=4.47 (0.012)	0.006	0.146
Amplitude Day	$F_{2,231}$ =3.72 (0.026, *0.031*)	$F_{2,231}$=15.7 (<0.0001, *0.119*)	$F_{4,231}$= 1.67 ns	0.995	0.042	$F_{2,237}$=3.30 (0.039)	0.742	0.143 0.032*
period	$F_{2,179}$ =3.53 (0.032, *0.038*)	$F_{2,179}$=0.29 ns	$F_{4,179}$= 0.02 ns	0.304	0.130 0.023*	$F_{2,185}$=3.52 (0.032)	0.326	0.298 0.024*
power	$F_{2,231}$ =2.76 (0.065, *0.023*)	$F_{2,231}$=40.7 (<0.0001, *0.260*)	$F_{4,231}$= 0.16 ns	0.056	0.911	$F_{2,237}$=5.77 (0.004)	0.005	0.87
2022	Two-way ANOVA, *YFPi v Epbmal1i*							
MI	$F_{1,174}$ =4.77 (0.03, *0.027*)	$F_{3,174}$=1.32 ns	$F_{3,174}$=0.63 ns					
Amplitude Day + Night	$F_{1,174}$ =0.42 ns	$F_{3,174}$=8.24 (<0.0001, *0.124*)	$F_{3,174}$=0.51 ns					
Amplitude Night	$F_{1,174}$ =6.35 (0.013, *0.035*)	$F_{3,174}$=8.09 (<0.0001, *0.122*)	$F_{3,174}$=0.39 ns					
Amplitude Day	$F_{1,174}$ =1.03 ns	$F_{3,174}$=3.29 (0.022, *0.054*)	$F_{3,174}$=0.49 ns					
period	$F_{1,136}$ =0.40 ns	$F_{3,136}$=3.57 (0.016, *0.073*)	$F_{3,136}$=0.78 ns					
power	$F_{1,174}$ =5.36 (0.022, *0.030*)	$F_{3,174}$=4.28 (0.006, *0.069*)	$F_{3,174}$=0.45 ns					
	Two-way ANOVA, *all 3 knockdowns*					One-way ANOVA – *all 3 knockdowns*		

MI	$F_{2,119}$ =5.74 (0.004, *0.088)*	$F_{1,119}$=10.37 (0.002, *0.080)*	$F_{2,119}$=1.39 ns	0.072	0.003	$F_{2,208}$=4.08 (0.018)	0.056	0.029
Amplitude Day + Night	$F_{2,119}$ =0.03 ns	$F_{1,119}$=0.90 ns	$F_{2,119}$=0.39 ns	0.98	0.99	$F_{2,208}$=1.25 ns	-	-
Amplitude Night	$F_{2,119}$ =3.56 (0.032, *0.056)*	$F_{1,119}$=16.5 (<0.0001, *0.122)*	$F_{2,119}$=0.49 ns	0.22	0.02	$F_{2,208}$=4.15 (0.017)	0.009	0.48
Amplitude Day	$F_{2,119}$ =1.51 ns	$F_{1,119}$=1.07 ns	$F_{2,119}$=0.88 ns	-	-	$F_{2,208}$=2.07 ns	-	-
period	$F_{2,100}$ = 0.46 ns	$F_{1,100}$=0.09 ns	$F_{2,119}$=0.05 ns	-	-	$F_{2,167}$=0.89 ns	-	-
power	$F_{2,119}$ =1.15 ns	$F_{1,119}$=9.55 (0.003, *0.074)*	$F_{2,119}$=0.23 ns	0.54	0.25	$F_{2,209}$=3.23 (0.041)	0.024	0.45

S1 Table. Results of behavioural ANOVAs.

Red denotes significant effects, blue, marginal ones. P and η2 (effect size, *italics*) values are provided for the significant/marginally significant variables in the two-way ANOVAs. Asterisks denote where the ANOVA Knockdown factor was significant but the Dunnett's test comparing each of *Epbmal1i* and *Epcry2i* with control *YFPi* was not. The significant effect was generated by the *Epbmal1i v Epcry2i* comparison as detected with a Tukey test. In 2022, two-way ANOVAs were performed for the four collections that contained only *YFPi* and *Epbmal1i* animals, so no *post-hoc* tests are required. There were two collections in which all three knockdown groups were compared and *post-hoc* results are provided for the two-way ANOVA. The 2022 one-way ANOVA pools all the data from all four collections with the three knockdown groups. *Post-hoc* tests were performed only when there was a significant variable in the ANOVA.
