# Supplementary material for: The circadian clock gene bmal1 is necessary for co-ordinated circatidal rhythms in the marine isopod Eurydice pulchra (Leach)
Source: PLoS Genet. 2023 Oct 19;19(10):e1011011. doi: 10.1371/journal.pgen.1011011 (PMC10617734; doi:10.1371/journal.pgen.1011011)
Supplement: S2 Table — (PDF) [file pgen.1011011.s006.pdf]

SEASON	MI			Day +Night amplitude			Night amplitude			Day amplitude		
2016	WT^{YFPi}	*Epbmal1i*	*Epcry2i*	WT^{YFPi}	*Epbmal1i*	*Epcry2i*	WT^{YFPi}	*Epbmal1i*	*Epcry2i*	WT^{YFPi}	*Epbmal1i*	*Epcry2i*
early	0.48	0.31	0.09	2.16	1.94	2.27	2.43	2.01	2.27	1.87	1.86	2.25
mid	0.47	0.18	0.05	1.95	1.80	1.98	2.08	1.80	1.92	1.78	1.80	2.08
late	0.19	0.22	0.37	1.61	1.57	1.58	1.60	1.59	1.47	1.59	1.56	1.53
2022												
early	1.20	0.77		1.42	1.35		1.98	1.68		0.78	0.96	
mid	0.92	0.51	0.01	1.62	1.71	1.69	2.01	1.90	1.66	1.17	1.43	1.72
late	1.14	.87	0.84	1.81	1.75	1.72	2.33	2.14	2.12	1.23	1.34	1.29
autumn	0.79	0.79		1.55	1.42		1.91	1.79		1.13	1.03	

Circatidal period (h)			Circatidal power			Arrhythmicity %		
WT^{YFPi}	*Epbmal1i*	*Epcry2i*	WT^{YFPi}	*Epbmal1i*	*Epcry2i*	WT^{YFPi}	*Epbmal1i*	*Epcry2i*
12.37	12.55	12.03	1.87	1.69	1.8	0	9.1	0
12.35	12.68	12.27	1.85	1.62	1.77	0	22.7	5
12.43	12.53	12.22	1.20	1.10	1.26	25.9	49.1	31.0
12.06	12.14		1.72	1.36		22.7	33.3	
12.3	12.27	12.16	1.53	1.48	1.33	13.6	35.3	30.0
12.31	12.27	12.23	1.94	1.73	1.7	3.3	19.2	5.0
12.34	12.58		1.57	1.37		15.0	33.5	

S2 Table. Mean values for circatidal parameters for each collection per season.
