# Supplementary material for: The circadian clock gene bmal1 is necessary for co-ordinated circatidal rhythms in the marine isopod Eurydice pulchra (Leach)
Source: PLoS Genet. 2023 Oct 19;19(10):e1011011. doi: 10.1371/journal.pgen.1011011 (PMC10617734; doi:10.1371/journal.pgen.1011011)
Supplement: S3 Table — (PDF) [file pgen.1011011.s007.pdf]

S3 Table. Description of spectral and autocorrelogram plots from S3 and S4 Figs

The power of the circatidal peak at the circatidal and circalunidian periods (~12.5 and ~25 h) is shown and the amplitude of the autocorrelogram at each cycle is represented as peak R value minus trough.

Collection 2016	Knockdown	Circatidal spectral power	Circalunidian spectral power	Autocorrelation peak - trough value			
				1st cycle	2nd cycle	3rd cycle	4th cycle
Early summer	*YFPi*	3.47	-	1.03	0.89	0.66	0.48
	Epbmal1i	2.97	-	0.92	0.75	0.53	0.34
	Epcry2i	3.04	-	1.03	0.84	0.58	0.36
Mid-summer	*YFPi*	3.49	1.18	0.77	0.65	0.43	0.50
	Epbmal1i	1.61	1.97	0.17	0.22	0.06	0.12
	Epcry2i	2.61	-	0.53	0.58	0.35	0.36
Late summer	*YFPi*	1.46	-	0.47	0.27	0.12	0.07
	Epbmal1i	1.5	-	0.29	0.27	0.13	0.05
	Epcry2i	1.68	-	0.27	0.2	0.14	0.01
2022							
Early summer	*YFPi*	1.74	4.28	0.97	0.77	-	-
	Epbmal1i	1.29	1.93	0.29	0.13	-	-
Mid-summer	*YFPi*	1.37	1.77	0.25	0.38	0.06	0.04
	Epbmal1i	1.71	3.27	0.73	0.57	-	-
	Epcry2i	1.89	1.41	0.42	0.50	0.15	0.24
Late summer	*YFPi*	2.67	1.84	0.46	0.75	0.3	0.43
	Epbmal1i	2.46	1.71	0.52	0.63	0.25	0.36
	Epcry2i	2.27	1.67	0.42	0.71	0.26	0.39
Autumn	*YFPi*	2.91	-	0.23	0.52	0.31	0.36
	Epbmal1i	1.42	1.25	0.07	0.13	0.05	0.11
