# Supplementary material for: The circadian clock gene bmal1 is necessary for co-ordinated circatidal rhythms in the marine isopod Eurydice pulchra (Leach)
Source: PLoS Genet. 2023 Oct 19;19(10):e1011011. doi: 10.1371/journal.pgen.1011011 (PMC10617734; doi:10.1371/journal.pgen.1011011)
Supplement: S4 Table — (PDF) [file pgen.1011011.s008.pdf]

S4 Table. Primers for dsRNAi and qRT-PCR

	**Primer name**	**Forward sequence (5’→3’)**	**Reverse sequence (5’→3’)**
dsRNAi	*EpClk dsRNA*	*T7-CTTACTCCGTACCGCTCGTC*	*T7-GCTGCTGTTGCATGTGACTT*
	Epbmal1_1 dsRNA	*T7-GGTATCCGATTGTGCGTCTT*	*T7-AGAAGTCGACAGGTTCCCCT*
	Epbmal1_2 dsRNA	*T7-CCGTTCCAAAAGACACCACT*	*T7-ACTCTGGTCTCCTGTGTGGG*
	Epcry2 dsRNA	*T7-TCCAGATTCCGTGGGATAAGA*	*T7-CTCTCTTGTCCTTCTTCGGG*
	yfp dsRNA	*T7-AGGACGACGGCAACTACAAG*	*T7-GTCCATGCCGAGAGTGATCC*
qRT-PCR	*EpClk qPCR (122bp)*	*GCAACAGCAGACCTTCCTTC*	*GGGTGAGAGGGTTGAGAGGT*
	Epbmal1 qPCR (131bp)	*CTCTTCGTCGTTGGTTGTGA*	*GCCAGATCTTTCGGATGAAG*
	Epcry2 qPCR (100bp)	*ACCTGCCCAATCCTGTGTGG*	*GGCCTCCCAAACGAAGCTACC*
	EpRPL32 qPCR (107bp)	*CAAAATTGGAGGAAGCCAAA*	*TGCTTTGTTTTCTTGGCTGA*
	Eptim3 qPCR (120bp)	*AGCTGAATTTCCACCTCTGCG*	*CCAGAGTCGCGTTCCTCTTC*

T7- TAATACGACTCACTATAGGGAG(A)
